# *Snhg18* promotes hypoxic pulmonary hypertension by enhancing glycolysis

**DOI:** 10.1186/s12931-026-03707-1

**Published:** 2026-05-18

**Authors:** Tianyu Qu, Kai Ma, Qiang Du, Chun Zhang, Ziling Shen, Enze Wang, Zhixuan Chen, Pingsheng Chen, Yingying Liu, Ruifeng Zhang

**Affiliations:** 1 Department of Respiratory Medicine, Zhongda Hospital, School of Medicine, Southeast University, 87 Dingjiaqiao, Nanjing, Jiangsu 210009 China; 2 Department of Respiratory and Critical Care Medicine, Suzhou Municipal Hospital, Gusu School, The Affiliated Suzhou Hospital of Nanjing Medical University, Nanjing Medical University, 242 Guangji Road, Soochow, Jiangsu 215000 P.R. China; 3 Department of Tuberculosis, The Second Hospital of Nanjing, Nanjing University of Chinese Medicine, Nanjing, 210009 China; 4 Department of Pathology, School of Medicine, Southeast University, Nanjing, China

**Keywords:** Long non-coding RNA, *Snhg18*, Metabolism, Pulmonary hypertension

## Abstract

**Background:**

Pulmonary hypertension (PH) is a life-threatening vascular disorder characterized by progressive pulmonary vascular remodeling. Emerging evidence has indicated that long non-coding RNAs (lncRNAs) play vital roles in the pathogenesis of PH. In this study, we aim to clarify the role of lncRNA small nucleolar RNA host gene 18 (*Snhg18)* in pulmonary vascular remodeling and investigate its underlying mechanisms.

**Methods:**

Differential gene expression was detected by qRT-PCR and Western blot assays. CCK-8, EdU, transwell assays, and the in vivo pulmonary hypertension model were used to investigate the effect of *Snhg18* on pulmonary hypertension. Dual-luciferase reporter assay, chromatin immunoprecipitation (ChIP) assay, RNA pull-down, and transcriptome sequencing were employed to explore the mechanism by which *Snhg18* functions in pulmonary hypertension.

**Results:**

LncRNA *Snhg18* is upregulated in pulmonary artery smooth muscle cells (PASMCs) and lung tissues under hypoxic conditions. Inhibition of *Snhg18* attenuates cell proliferation in vitro and in vivo. Mechanistically, copy number amplification promotes the expression of *Snhg18* at the genomic level, and the transcription factor Sp1 activates *Snhg18* transcription by binding to its promoter. Furthermore, *Snhg18* interacts with the m6A “reader” protein heterogeneous nuclear ribonucleoprotein A2/B1 (Hnrnpa2b1), thus increasing the stability of enolase 3 (Eno3) mRNA in an m6A-dependent manner. The elevation of Eno3 augments glycolysis in PASMCs, thus promoting cell proliferation and vascular remodeling.

**Conclusions:**

Our study demonstrates the important role of *Snhg18*/Hnrnpa2b1/Eno3 axis in pulmonary vascular remodeling, and provides a potential novel therapeutic target for PH.

**Supplementary Information:**

The online version contains supplementary material available at 10.1186/s12931-026-03707-1.

## Background

Pulmonary hypertension (PH) is a progressive pulmonary vascular disease characterized by elevated mean pulmonary artery pressure and sustained pulmonary vascular remodeling, ultimately resulting in right ventricular failure and even death [1, 2]. In addition to pulmonary artery endothelial dysfunction and a series of pathological alterations, the hyperproliferation of PASMCs is considered a major contributor to pulmonary vascular remodeling [3, 4]. Hypoxia is an important trigger of excessive PASMC proliferation during the remodeling process [5], and targeted inhibition of hypoxia-induced PASMC proliferation represents a potential effective therapeutic strategy for PH.

LncRNAs (Long noncoding RNAs) play crucial roles in gene expression regulation at epigenetic, transcriptional, and post-transcriptional levels [6]. It has been reported that lncRNAs contribute to the pathogenesis of PH. For example, Liu et al. demonstrated that lncRNA *VELRP* could promote H3K4me3 modification of CDKs by binding to WDR5, thereby facilitating PASMC proliferation [7]. Knockdown of lncRNA *Tug1* diminishes pulmonary remodeling by inhibiting Foxc1-mediated PASMC proliferation and migration [8]. Hence, further exploration of the regulatory mechanisms of lncRNAs in PH could enhance our knowledge of the pathogenesis of PH. The small nucleolar RNA host gene (Snhg) family represents an important group of lncRNAs, and many members of this family have been reported to promote cell proliferation and the development of various tumors [9–11]. Additionally, the family members can also play important roles in vascular remodeling and PH development. For example, *SNHG11* promotes cell proliferation by increasing PRPF8 protein stability, thus promoting PH progression [12]. As a member of this family, *Snhg18* has been reported to regulate the miR-211-5p/BRD4 axis to facilitate tumorigenesis of lung cancer [13]. Given that pulmonary hypertension exhibits certain tumor-like behaviors [14], this suggests a potential role for *Snhg18* in PH pathogenesis. Therefore, we sought to study *Snhg18* in PH.

N6-methyladenosine (m6A) is the most prevalent internal RNA modification in eukaryotes, regulated by writer, reader, and eraser proteins [15]. An increasing number of studies suggest that m6A modification is closely associated with various biological processes, including the pathogenesis of PH [16, 17]. In our previous study, as the core catalytic enzyme subunit of the m6A writer complex, RNA methyltransferase METTL3 accelerates the progression of PH by increasing the m6A modification level of RBPJ in PASMCs [18]. However, whether lncRNAs could regulate gene expression in PASMCs via m6A modification in the pathogenesis of PH remains unclear.

An increasing number of studies have focused on the role of cellular metabolic remodeling in the development of PH. Glucose metabolism is one of the major metabolic pathways that cells rely on. Compared to mitochondrial oxidative phosphorylation, which produces more ATP, rapidly proliferating cells favor glycolysis, as it supplies more precursors for biosynthesis [19]. Enhanced glycolytic capacity in PASMCs has been implicated in promoting pulmonary vascular remodeling [20, 21]. However, the specific regulatory effect of lncRNAs on glycolysis in PASMCs warrants further investigation.

In this study, we identified the role of lncRNA *Snhg18* in pulmonary vascular remodeling of PH. The increased expression of *Snhg18* is activated by copy number amplification and Sp1-mediated transcriptional regulation. Moreover, we demonstrated that *Snhg18* binds to Hnrnpa2b1 and maintains the mRNA stability of Eno3 in an m6A-dependent manner, thereby enhancing glycolysis and promoting PH progression. Collectively, these findings suggest that Snhg18 may serve as a novel target for PH treatment.

## Methods

### Human plasma collection

The plasma samples from healthy controls and patients with pulmonary hypertension secondary to chronic obstructive pulmonary disease (COPD-PH) were obtained at Zhongda Hospital of Southeast University. COPD was diagnosed according to the Global Initiative for Chronic Obstructive Lung Disease (GOLD) guideline [22], and pulmonary hypertension was defined as systolic pulmonary artery pressure (sPAP) > 35mmHg [23, 24]. After centrifugation at 1500 g for 15 min at 4℃, the plasma was separated from the whole blood and subsequently stored at -80℃ until further extraction. The clinical characteristics of the patients are provided in Table S2.

### Establishment of animal models

Male C57BL/6 mice (6–8 weeks old) were purchased from Changzhou Cavens Laboratory Animal Co. Ltd. To construct a hypoxic PH model, mice were housed in a hypoxic chamber (10% O_2_) for 3 weeks, whereas the control mice were housed in normoxic condition. To investigate the effect of *Snhg18* inhibition on hypoxic PH progression, mice were anesthetized with tribromoethanol via intraperitoneal injection (0.35 mg/g), and intratracheally administered 1 × 10^11^ vector genomes of adeno-associated virus 9 (AAV9) vector containing the short-hairpin RNA (shRNA) targeting *Snhg18* (sh*Snhg18*) or negative control (shNC) in a final solution of 100 µl, and 4 weeks later exposed to 10% oxygen for 3 weeks to induce PH. To generate the *Snhg18* overexpression model, mice were anesthetized and intratracheally administered 1 × 10^11^ vector genomes of adeno-associated virus 9 (AAV9) vector containing full-length *Snhg18* or negative control (Vector) in a final solution of 100 µl. Four weeks later, DCA (80 mg/kg/d, 100 µl) or saline was daily given to mice for three weeks by gavage [25]. The AAV vectors were purchased from GeneChem, and the sequence of sh*Snhg18* is provided in Table S3.

### Right ventricular systolic pressure (RVSP) and right ventricular hypertrophy (RVH) assessments

For RVSP assessment, mice were anesthetized with isoflurane inhalation (2.0%) during the process. We first made a small incision in the mouse abdominal wall and visualized the diaphragm through the incision. Then, we inserted a 25-gauge needle connected to a pressure transducer through the diaphragm into the right ventricle to measure RVSP [26–28]. The data were recorded and analyzed using the PowerLab system and LabChart software (ADInstruments). The mice were euthanized by exsanguination under anesthesia with isoflurane inhalation (2.0%). After cardiac isolation, the right ventricle was carefully separated from the left ventricle (LV) and septum (S), then weighed to calculate the RV hypertrophy index as RV/(LV + S).

### Echocardiography

Echocardiography was performed in a blinded manner by the Animal Core Facility of Nanjing Medical University using VisualSonics Vevo 3100. The analysis of velocity time integral (VTI), pulmonary artery acceleration time (PAT), and pulmonary artery ejection time (PET) was performed with VevoLab software (VisualSonics).

### Histological analysis and immunofluorescence (IF) staining

The fixed lung tissues were embedded in paraffin and sectioned into 5 μm slices. Then the sections were subjected to hematoxylin and eosin (H&E) staining to determine the thickness of the vascular walls. For each sample, around 20 pulmonary arteries were chosen in a randomized and blinded manner for assessment. For immunofluorescence staining, the sections were subjected to antigen retrieval after deparaffinization and rehydration. Following blocking with 1% BSA, the lung sections were stained with the specific primary antibodies at 4℃. The next day, the sections were incubated with Alexa Fluor-conjugated secondary antibodies for 2 h at room temperature. DAPI was used to stain nuclei. The VWF antibody (27186-1-AP, Dilution ratio, 1:200), α-SMA antibody (67735-1-Ig, Dilution ratio, 1:400), PCNA antibody (10205-2-AP, Dilution ratio, 1:200), SP1 antibody (21962-1-AP, Dilution ratio, 1:200), HNRNPA2B1 antibody (14813-1-AP, Dilution ratio, 1:400), and ENO3 antibody (55234-1-AP, Dilution ratio, 1:200) were from Proteintech. The muscularization of vessels was assessed based on the muscular coat surrounding the endothelium. Vessels were classified as non-muscularized (no apparent muscle), partially muscularized (with a crescent of muscle), or fully muscularized (with a complete ring of muscle) [29].

### Fluorescent in situ hybridization (FISH) assays

The fixed lung sections or cells were hybridized in a prehybridization buffer for 30 min. Then they were hybridized with the *Snhg18* probe in a hybridization buffer at 37℃ overnight. After washing with saline sodium citrate, the lung sections or cells were stained with DAPI for 10 min. The Cy3-labeled *Snhg18* probes were synthesized by RiboBio.

### RNA extraction, DNA extraction and qRT-PCR analysis

Total RNA was extracted from cells or lung tissues using TRIzol reagent (Invitrogen). For each sample, 1 µg RNA was used to reverse transcribe into cDNA using HiScript III RT SuperMix for qPCR (+ gDNA wiper) (Cat. R323-01, Vazyme). qRT-PCR analysis was performed with Taq Pro Universal SYBR qPCR Master Mix (Cat. Q712-02, Vazyme) according to the manufacturer’s protocol. Results were normalized to the expression of *β-actin*. The DNA in cells and lung tissues was isolated using the TIANamp Genomic DNA kit (Cat. DP304, TIANGEN) according to the manufacturer’s protocol. The DNA in the plasma of patients and controls was isolated using the Serum/Plasma Circulating DNA kit (Cat. DP339, TIANGEN) according to the manufacturer’s protocol. The primer sequences are provided in Table S3.

### Cell culture

Mouse pulmonary artery smooth muscle cells (mPASMCs) were obtained from Procell Life Science & Technology and cultured in Dulbecco’s Modified Eagle Medium (DMEM) supplemented with 10% fetal bovine serum (FBS). All mPASMCs at passages 3–5 were used in the experiments. Human pulmonary artery smooth muscle cells (HPASMCs) were obtained from ScienCell Research Laboratories and cultured in smooth muscle cell medium (ScienCell Research Laboratories). All HPASMCs at passages 4–6 were used in the experiments. Mouse pulmonary artery endothelial cells (mPAECs) were obtained by Procell Life Science & Technology and cultured in endothelial cell medium (ScienCell Research Laboratories). The cells were subjected to hypoxic treatment using a tri-gas incubator (Thermo Fisher) with an atmosphere containing 3% O2, 5% CO2, and 92% N2. The purification of PASMCs was identified by immunofluorescence staining for α-SMA.

### Isolation of cytoplasmic and nuclear RNA

The separation of the nuclear and cytosolic fractions was performed using the Cytoplasmic & Nuclear RNA Purification Kit (Cat. 21000, Norgen Biotek) according to the manufacturer’s protocol.

### Cell transfection

To knock down the target genes in cells, small interfering RNAs (siRNAs) were transfected into cells using Lipofectamine 3000 (Invitrogen), including the corresponding controls. The RNA interference oligonucleotide sequences are provided in Table S3. For the overexpression assays, the sequences of target genes were subcloned into a pcDNA3.1 vector, and the empty vector was used as the control. The plasmids were transfected into cells using Lipofectamine 3000 (Invitrogen).

### Cell counting kit-8 (CCK-8) assays, EDU assays, and transwell assays

In the CCK-8 assays, the transfected cells were plated in a 96-well plate at a density of 5 × 10^3^ cells/well. Cells were treated under hypoxic (3% O2) or normoxic (21% O2) conditions for 24 h. Then, 10 µL Cell Counting Kit-8 reagent (Cat. B34304, Selleck) was added to each well. After incubation at 37 °C for 2 h, the absorbance at 450 nm was determined using a microplate reader. To conduct the EDU assay, the Cell-Light EdU Apollo567 In Vitro Kit (Cat. C10310-1, RiboBio) was used according to the protocol to determine cell proliferation. The transwell assay was performed using 8 μm transwell chambers (Cat. 3422, Corning). Approximately 1 × 10^4^ transfected cells suspended in serum-free medium were added to the upper chamber, with medium containing 20% fetal bovine serum placed in the lower chamber. After 24 h, the cells were fixed with 4% paraformaldehyde and stained with crystal violet.

### Western blot assays

Protein from cultured cells or lung tissues was extracted using RIPA lysis buffer. Protein lysates were separated by 4–20% SDS-PAGE gels and transferred to a 0.45 μm PVDF membrane (Millipore). After blocking with 5% skim milk, the membranes were incubated with specific primary antibodies at 4℃. The next day, the membranes were incubated with the corresponding secondary antibodies. Immunoblots were detected using the Tanon luminescent imaging system, and Image J software was utilized for quantification. β-actin antibody (66009-1-Ig, Dilution ratio, 1:10000, Proteintech) was used as a control. PCNA antibody (10205-2-AP, Dilution ratio, 1:5000), CDK2 antibody (10122-1-AP, Dilution ratio, 1:5000), P27 antibody (25614-1-AP, Dilution ratio, 1:1000), SP1 antibody (21962-1-AP, Dilution ratio, 1:2000), HNRNPA2B1 antibody (14813-1-AP, Dilution ratio, 1:5000), and ENO3 antibody (55234-1-AP, Dilution ratio, 1:2000) were from Proteintech. METTL3 antibody (A19079, Dilution ratio, 1:1000) was from ABclonal. HIF-1α antibody (ab179483, Dilution ratio, 1:1000) was from Abcam.

### Chromatin immunoprecipitation (ChIP) assays

The ChIP assays were conducted using the ChIP Assay Kit (Cat. P2080S, Beyotime). The SP1 antibody was obtained from Proteintech (21962-1-AP), and normal rabbit IgG antibody was used as the control. The enrichment of the immunoprecipitated DNA was determined by qRT-PCR analysis. The ChIP primer sequences are provided in Table S3.

### Dual-luciferase reporter assays

The dual-luciferase reporter assay was conducted using the Dual-Luciferase Reporter Assay System Kit (Cat. E1910, Promega) according to the manufacturer’s protocol. The cells were co-transfected with the constructed luciferase vector and the Renilla luciferase plasmid. Luciferase activity was determined by normalizing the luminescence value of firefly luciferase to that of the Renilla luciferase.

### In vitro transcription assays and RNA pull-down/mass spectrometry analysis

The indicated genes for the experiments were transcribed in vitro by using the mMESSAGE mMACHINE™ T7 Transcription Kit (Cat. AM1344, Invitrogen) according to the manufacturer’s protocol. Then RNA was subjected to desthiobiotinylation with the Pierce RNA 3’ End Desthiobiotinylation Kit (Cat.20163, Thermo Scientific), followed by the RNA pull-down assay using the Pierce Magnetic RNA-Protein Pull-Down Kit (Cat.20164, Thermo Scientific). The labeled RNA was then incubated with whole cell lysates from mPASMCs that had been cultured under hypoxia. Then eluted RNA-binding proteins were digested into peptides for mass spectrometry analysis or used for Western blot assays. Liquid chromatography-tandem mass spectrometry experiments were performed with a QExactive Orbitrap mass spectrometer (Thermo Finnigan).

### RNA immunoprecipitation (RIP) assays

The RIP assays were conducted using the RNA Immunoprecipitation Kit according to the manufacturer’s protocol (Cat. Bes5101, BersinBio). The HNRNPA2B1 antibody (14813-1-AP) was obtained from Proteintech. The enrichment of the immunoprecipitated RNA was determined by qRT-PCR analysis.

### Transcriptome sequencing analysis

After measuring the concentration of total RNA from transfected mPASMCs and control cells, the sample quality was evaluated using the Agilent 2200. The sequencing library of each RNA sample was prepared using the TruSeq Stranded mRNA Library Prep Kit (Illumina).

### RNA stability assays

The transfected cells were treated with actinomycin D (5 µg/ml, MedChemExpress) and cultured for the indicated time. After incubation for 0, 2, 4, and 6 h, the RNA samples were extracted and subjected to qRT-PCR analysis to evaluate the remaining RNA. Results were normalized to the expression of 18S rRNA.

### M6A dot blot assays

Total RNA extracted from the transfected cells was loaded on a nylon membrane (Millipore) installed in a BioDot apparatus (Bio-Rad), and then the membrane was crosslinked by ultraviolet light. After incubation with methylene blue, the membrane was blocked and incubated with the m6A antibody (202003) from Synaptic Systems overnight at 4℃.

### Methylated RNA immunoprecipitation (MeRIP) assays

The MeRIP assays were performed using the m6A Methylated RNA Immunoprecipitation Kit (Cat. Bes5203-2, BersinBio) according to the manufacturer’s protocol. The enrichment of the immunoprecipitated RNA was determined by qRT-PCR analysis.

### Glycolytic rate assays

The extracellular acidification rate (ECAR) was measured using the Seahorse XFe96 Extracellular Flux Analyzer (Agilent) with the Glycolytic Rate Assay Kit (Cat. 103344-100, Agilent), with the sequential addition of a mixture of antimycin A and rotenone (0.5 µM) and 2-DG (50 mM) to the system.

### Glucose consumption/lactate/pyruvate determination

To determine glucose consumption, the Glucose kit (Cat. A154-1-1, Nanjing Jiancheng Bioengineering Institute) was used to measure the remaining glucose levels in cell culture supernatants after the indicated treatments. The lactate level was determined using the Lactic Acid assay kit (Cat. A019-2-1, Nanjing Jiancheng Bioengineering Institute) following the manufacturer’s instructions. The Pyruvate assay kit (Cat. A081-1-1, Nanjing Jiancheng Bioengineering Institute) was used to measure the pyruvate concentration.

### Statistics

All data were presented as mean ± SD, and statistical analysis was performed using GraphPad Prism. The significance of differences between 2 groups was determined by Student’s t-test, while one-way ANOVA was performed for multiple comparisons. Two-sided *p*-values were calculated, with *p* < 0.05 considered significant.

## Results

### Hypoxia upregulates the expression of *Snhg18*

**Fig. 1:**
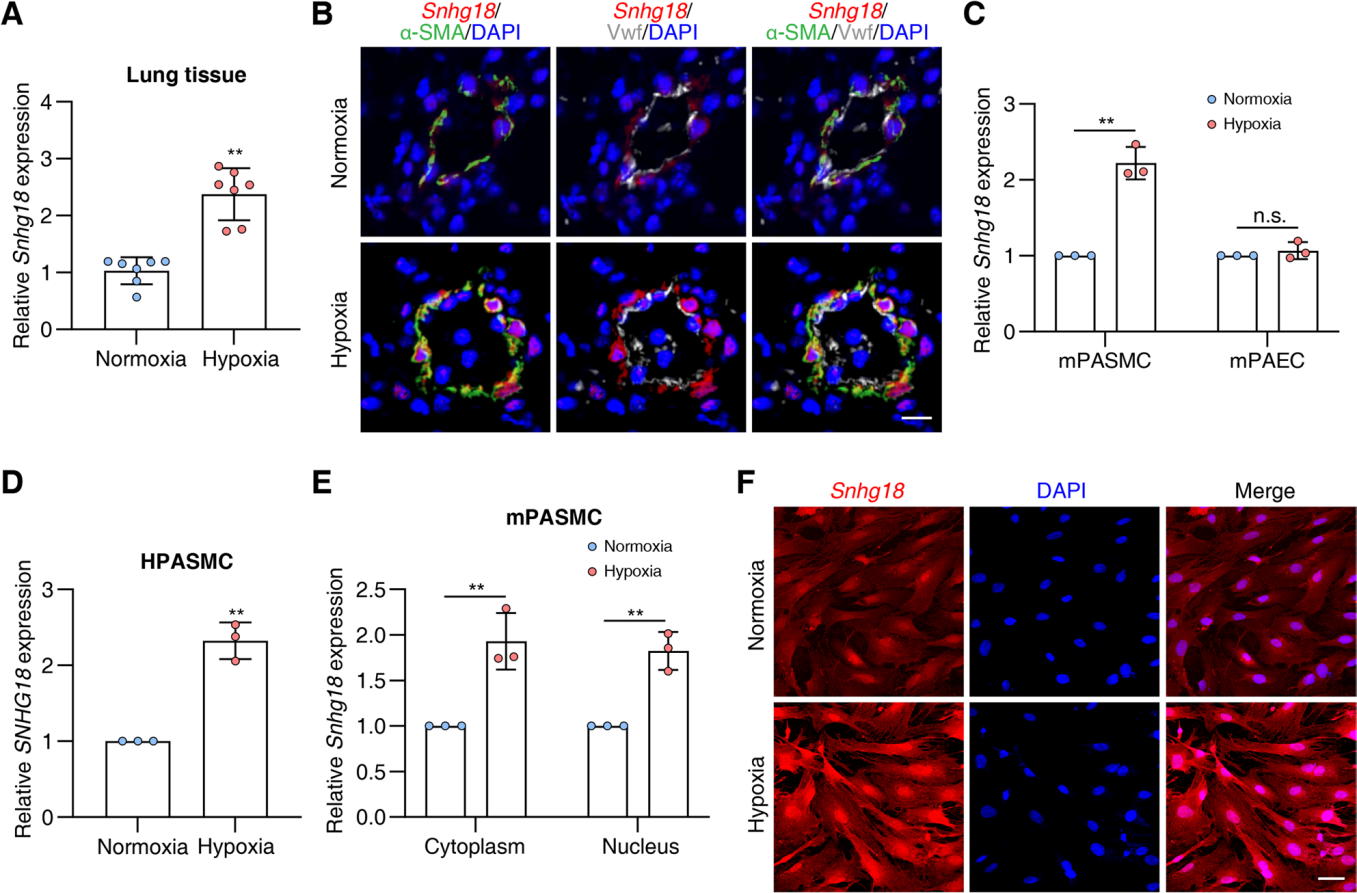
*Snhg18* expression is increased in lung tissues and PASMCs under hypoxic conditions. **A** qRT-PCR analysis of *Snhg18* expression in mouse lung tissues (*n* = 7). **B** Representative images of RNA FISH-IF staining of pulmonary arteries (PAs) for *Snhg18* (red), α-SMA (green), Vwf (white), and nuclei (blue). Scale bar 10 μm. **C** qRT-PCR analysis of *Snhg18* expression in cultured mPASMCs and mPAECs. **D** qRT-PCR analysis of *SNHG18* expression in HPASMCs. **E** After separation of nuclear and cytosolic fractions, *Snhg18* expression was measured by qRT-PCR in mPASMCs. **F** RNA FISH analysis of *Snhg18* expression in mPASMCs. Scale bar 50 μm. **A**, **C**, **D**, **E** The data were analyzed using two-tailed Student’s t-test. Data are presented as mean ± SD. **P < 0.01, n.s., not significant. DAPI indicates 4ʹ,6-diamidino-2-phenylindole

To determine the role of *Snhg18* in the development of hypoxia-induced pulmonary hypertension (HPH), we assessed *Snhg18* expression in the lung tissues of HPH mice using qRT-PCR analysis. The results showed that *Snhg18* was significantly elevated in hypoxic lung tissues compared to normoxic tissues (Fig. 1A). RNA FISH-IF analysis conducted on lung sections indicated that *Snhg18* was expressed in pulmonary arteries, with further confirmation of its hypoxia-induced upregulation (Fig. 1B). qRT-PCR analysis revealed a pronounced elevation of *Snhg18* in mouse pulmonary artery smooth muscle cells (mPASMCs) under hypoxic conditions, while no significant differences in expression were found in mouse pulmonary artery endothelial cells (mPAECs) (Fig. 1C). Similarly, increased expression of *SNHG18* was also confirmed in human pulmonary artery smooth muscle cells (HPASMCs) under hypoxia (Fig. 1D). Subsequent subcellular localization assays and cellular RNA FISH experiments demonstrated that *Snhg18* was upregulated in both the cytoplasm and the nucleus under hypoxia (Fig. 1E and F).

### *Snhg18* exacerbates hypoxic pulmonary hypertension by promoting PASMC proliferation

**Fig. 2:**
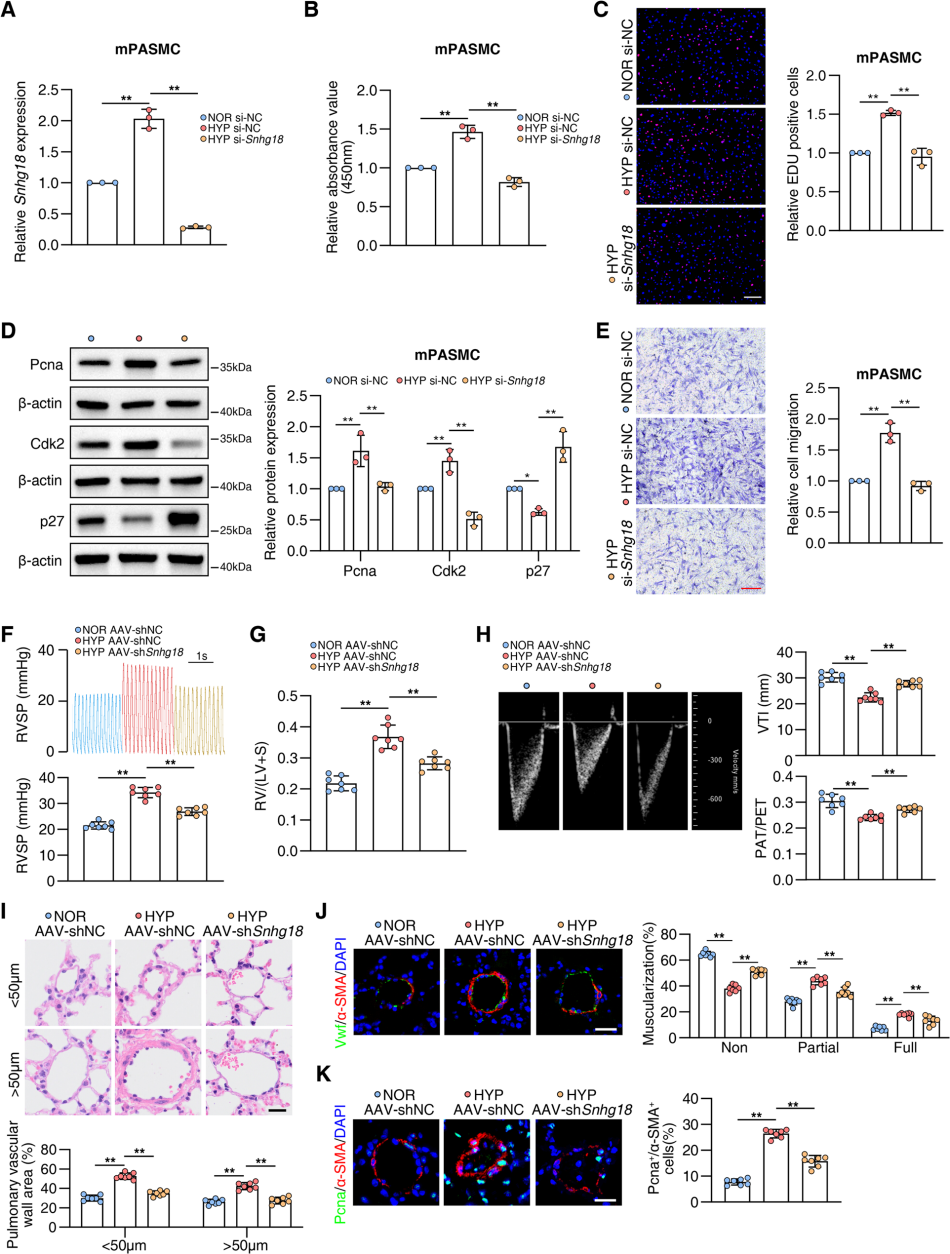
*Snhg18* exacerbates hypoxic pulmonary hypertension by promoting PASMC proliferation. **A** qRT-PCR analysis of *Snhg18* expression in mPASMCs after indicated treatments. **B** CCK8 assays determined the proliferation of mPASMCs after indicated treatments. **C** EDU assays determined the proliferation of mPASMCs after indicated treatments. Scale bar 200 μm. **D** Western blot analysis and quantification of Pcna, Cdk2, and p27 expression in mPASMCs. **E** Transwell assays determined the migratory ability of mPASMCs after indicated treatments. Scale bar 200 μm. **F-H** RVSP (**F**), the ratio of right ventricular (RV) to left ventricular (LV) plus septum (S) (**G**), the velocity time integral (VTI), and the ratio of pulmonary artery acceleration time to ejection time (PAT/PET) **H** in model mice (*n* = 7). **I** Representative images of H&E staining of pulmonary arteries (PAs) and the quantification of the vascular wall area index. Scale bar 20 μm. **J** Representative images of IF staining of PAs for Vwf (green), α-SMA (red), and nuclei (blue). The proportion of non, partially, or fully muscularized PAs was analyzed. Scale bar 20 μm. **K** Representative images of IF staining of PAs for Pcna (green), α-SMA (red) and nuclei (blue) and the quantification of Pcna^+^ cells in lung sections. Scale bar 20 μm. **A-K** The data were analyzed using one-way ANOVA with Dunnett’s multiple comparisons test. Data are presented as mean ± SD. *P < 0.05, **P < 0.01. NOR indicates normoxia; HYP, hypoxia; AAV, adeno-associated virus; RVSP, right ventricular systolic pressure; RV/(LV + S), right ventricular/ (left ventricle+septum); VTI, velocity time integral; PAT/PET, pulmonary artery acceleration time / pulmonary ejection time; and DAPI, 4ʹ,6-diamidino-2-phenylindole

To explore the function of *Snhg18* in the progression of HPH, we knocked down *Snhg18* in mPASMCs and HPASMCs with small interfering RNA (siRNA), and qRT-PCR analysis verified the silencing efficiency of siRNA (Fig. 2A and Figure S1A). Then CCK8 assays (Fig. 2B and Figure S1B) and EDU assays (Fig. 2C and Figure S1C) showed that hypoxia-induced proliferation of mPASMCs and HPASMCs was abolished by knockdown of *Snhg18*. Furthermore, Western blot assays showed that hypoxia significantly upregulated the expression of proliferating cell nuclear antigen (Pcna) and cyclin dependent kinase 2 (Cdk2) while downregulating p27, and knockdown of *Snhg18* could reverse these changes in both mPASMCs and HPASMCs (Fig. 2D and Figure S1D). Additionally, transwell assays demonstrated that *Snhg18* knockdown reduced the hypoxia-enhanced migratory capacity of both mPASMCs and HPASMCs (Fig. 2E and Figure S1E).

To further confirm the findings in vivo, we used AAV9 to repress *Snhg18* expression in mice. The mice were then subjected to hypoxia for 21 days to construct the HPH model. The efficiency of *Snhg18* knockdown was shown in Figure S2A and S2B. As expected, inhibition of *Snhg18* reduced the elevation of right ventricular systolic pressure (RVSP) and the weight ratio of right ventricular/ (left ventricle + septum) caused by hypoxia (Fig. 2F and G). Echocardiography further showed that inhibition of *Snhg18* ameliorated the reduction in pulmonary artery velocity time integral (VTI) and the ratio of pulmonary artery acceleration time to ejection time (PAT/PET) induced by hypoxia (Fig. 2H). In addition, we found that knockdown of *Snhg18* decreased the vascular wall thickness and the proportion of muscularized pulmonary arteries in HPH mice (Fig. 2I and J). Immunofluorescence staining for Pcna showed fewer proliferating mPASMCs in the pulmonary arteries after inhibition of *Snhg18* (Fig. 2K).

### DNA copy number amplification and Sp1-mediated transcriptional activation both contribute to upregulation of *Snhg18* in HPH

**Fig. 3:**
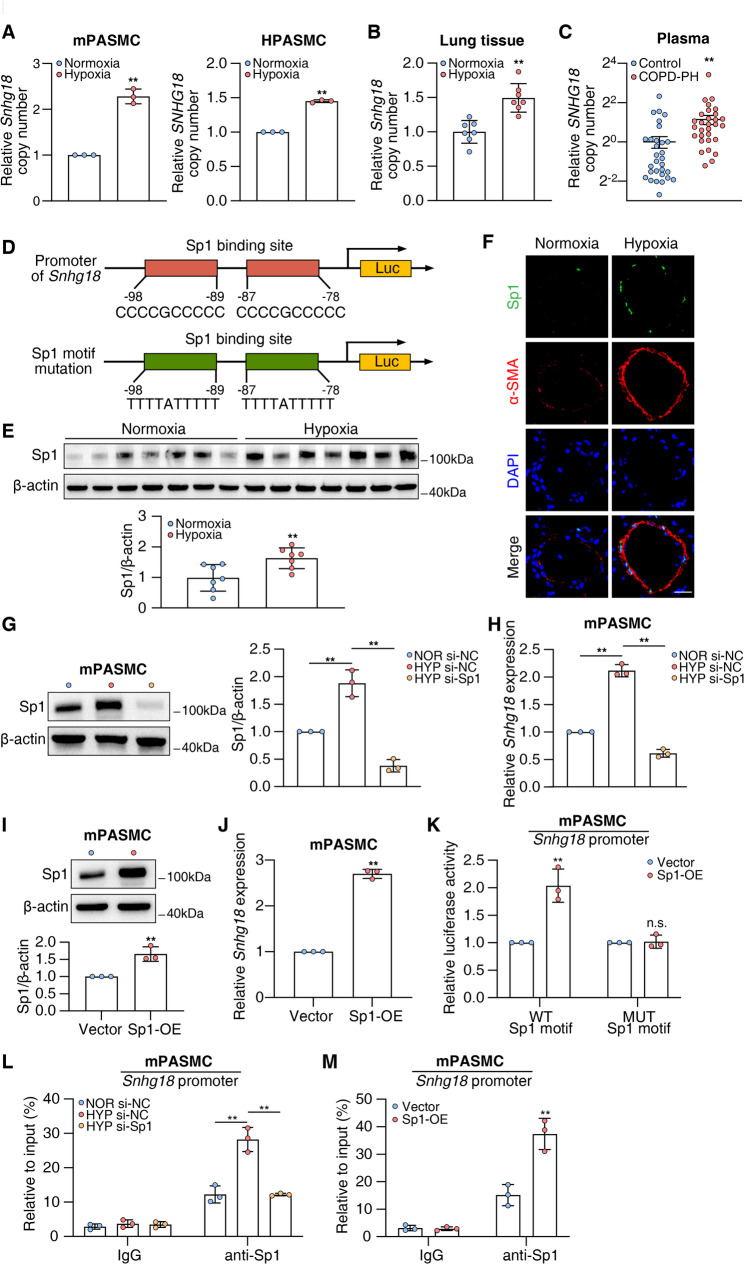
DNA copy number amplification and Sp1 upregulate *Snhg18* expression in hypoxia-induced PH. **A** qRT-PCR analysis of *Snhg18* DNA copy number in mPASMCs and HPASMCs. **B** qRT-PCR analysis of *Snhg18* DNA copy number in mouse lung tissues (*n* = 7). **C** qRT-PCR analysis of *SNHG18* DNA copy number in plasma from the healthy controls and COPD-PH patients (*n* = 30). **D** Schematic diagram of the *Snhg18* promoter with the putative Sp1 binding sites and the mutation information. **E** Western blot analysis and quantification of Sp1 expression in mouse lung tissues (*n* = 7). **F** Representative images of IF staining of PAs for Sp1 (green), α-SMA (red), and nuclei (blue). Scale bar 20 μm. **G** Western blot analysis and quantification of Sp1 expression in mPASMCs after indicated treatments. **H** qRT-PCR analysis of *Snhg18* expression in mPASMCs after indicated treatments. **I** Western blot analysis and quantification of Sp1 expression in mPASMCs after indicated treatments. **J** qRT-PCR analysis of *Snhg18* expression in mPASMCs after indicated treatments. **K** The dual-luciferase reporter assays detected the transcriptional activity of *Snhg18* with wild-type or mutated Sp1 motifs in the promoter. **L**, **M** ChIP assays detected the enrichment of Sp1 at the *Snhg18* promoter after Sp1 expression alteration. **A-C**, **E**, **I**-**K**, **M** The data were analyzed using a two-tailed Student’s t-test, (**G**, **H**, **L**) Data were analyzed using one-way ANOVA with Dunnett’s multiple comparisons test. Data are presented as mean ± SD. **P < 0.01. n.s., not significant. COPD-PH indicates pulmonary hypertension secondary to chronic obstructive pulmonary disease; DAPI, 4ʹ,6-diamidino-2-phenylindole; NOR, normoxia; HYP, hypoxia; OE, overexpression; WT, wild type; and MUT, mutation type

DNA copy number amplification (CNA) is a genetic alteration that often occurs in rapidly proliferating cells, such as tumor cells [30]. CNA often leads to increased transcript levels, while copy number deletion typically results in reduced gene expression. Previous studies have indicated that copy number variations contribute to the pathogenesis of PH [31–33]. Besides, substantial evidence suggests hypoxia acts as a driver of CNA [34, 35]. Therefore, we hypothesized that CNA could contribute to hypoxia-induced upregulation of *Snhg18* at the genomic level, thereby accelerating PASMC proliferation to promote HPH. qRT-PCR analysis in mPASMCs found that the copy number of *Snhg18* was elevated under hypoxic conditions, with a consistent trend observed in HPASMCs (Fig. 3A). In addition, we detected the copy number of *Snhg18* in lung tissues of HPH models and confirmed that the number of *Snhg18* copies in the hypoxia group was higher than that in the normoxia group (Fig. 3B). Meanwhile, elevated *SNHG18* copy number was also observed in plasma from patients with pulmonary hypertension secondary to chronic obstructive pulmonary disease (COPD-PH) compared with the controls (Fig. 3C).

Furthermore, we explored the reason for *Snhg18* upregulation at the transcriptional level and identified the Sp1 binding site in the promoter of the *Snhg18* gene using the JASPAR database (Fig. 3D). Previous studies have shown that Sp1 contributes to PASMC proliferation [36, 37]. Hence, we wondered whether Sp1 mediates the upregulation of *Snhg18*. Western blot assays were performed to detect Sp1 expression in HPH models. As shown in Fig. 3E, the expression of Sp1 was significantly elevated in the hypoxia group rather than the normoxia group. Immunofluorescence staining of pulmonary arteries further confirmed that hypoxia upregulated Sp1 expression (Fig. 3F). Importantly, after knockdown of hypoxia-induced Sp1 elevation (Fig. 3G and Figure S3A), *Snhg18* expression was significantly reduced in both mPASMCs and HPASMCs (Fig. 3H and Figure S3B). On the contrary, overexpression of Sp1 induced *Snhg18* expression in both mPASMCs and HPASMCs (Fig. 3I and J, Figure S3C and S3D). Next, luciferase reporter assays were conducted to measure the activity of the *Snhg18* promoter. We found that overexpression of Sp1 increased the luciferase activity of the wild-type *Snhg18* promoter, whereas mutation of the Sp1 binding site abolished this effect (Fig. 3K). ChIP-qPCR assays demonstrated that hypoxia increased the binding between Sp1 and the promoter of *Snhg18*, knockdown of Sp1 weakened this occupancy, and Sp1 overexpression enhanced the binding (Fig. 3L and M).

### *Snhg18* interacts with Hnrnpa2b1 in PASMCs

**Fig. 4:**
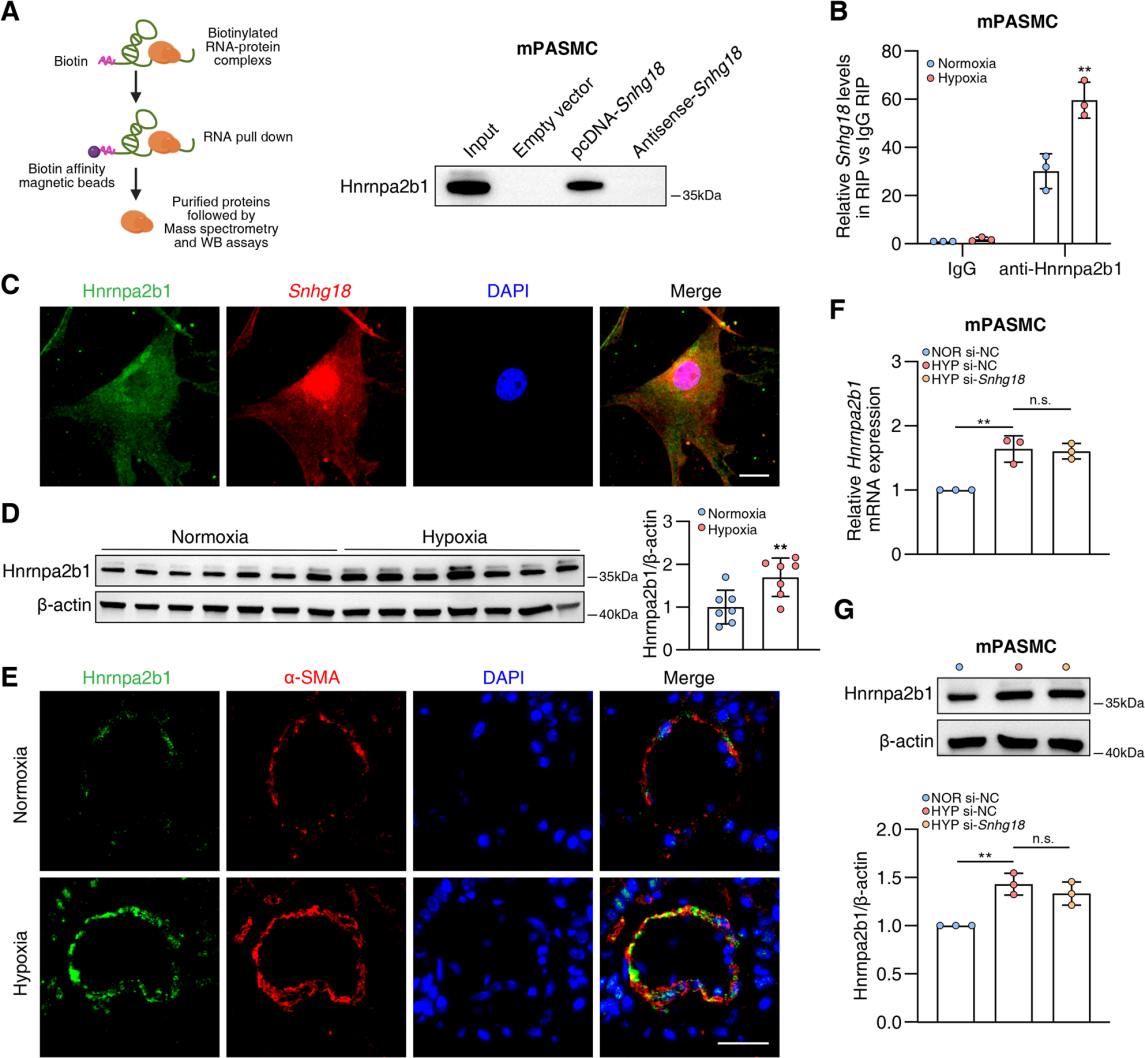
*Snhg18* directly binds to Hnrnpa2b1. **A** The RNA pull-down assays followed by mass spectrometry analysis and Western blot analysis revealed the interaction between *Snhg18* and Hnrnpa2b1 in mPASMCs. **B** The RIP assays detected the interaction between *Snhg18* and Hnrnpa2b1 in mPASMCs. **C** The co-localization analysis of *Snhg18* and Hnrnpa2b1 in mPASMCs. *Snhg18* was detected by the RNA FISH assay, and Hnrnpa2b1 was detected by IF staining. Scale bar 20 μm. **D** Western blot analysis and quantification of Hnrnpa2b1 expression in mouse lung tissues (*n* = 7). **E** Representative images of IF staining of PAs for Hnrnpa2b1 (green), α-SMA (red), and nuclei (blue). Scale bar 20 μm. **F** qRT-PCR analysis of *Hnrnpa2b1* expression in mPASMCs. **G** Western blot analysis and quantification of Hnrnpa2b1 expression in mPASMCs. **B**,** D** The data were analyzed using a two-tailed Student’s t-test, (**F**,** G**) The data were analyzed using one-way ANOVA with Dunnett’s multiple comparisons test. Data are presented as mean ± SD. **P < 0.01. n.s., not significant. DAPI indicates 4ʹ,6-diamidino-2-phenylindole; NOR, normoxia; and HYP, hypoxia

Since lncRNAs usually exert their functions by interacting with specific proteins [38], we performed RNA pull-down assays followed by mass spectrometry to identify the protein interacting with *Snhg18* (Fig. 4A). Among the functionally annotated proteins (Table S1), we verified Hnrnpa2b1 as the *Snhg18*-binding protein. Hnrnpa2b1 attracted our attention because of its role in promoting cell proliferation [39], and it has been reported to maintain mRNA stability at the post-transcriptional level in the cytoplasm [40]. Western blot assays showed that Hnrnpa2b1 was detected in *Snhg18* pull-down protein complexes but not in empty vector or antisense-*Snhg18* pull-down protein complexes (Fig. 4A). Furthermore, RIP assays confirmed that the interaction between *Snhg18* and Hnrnpa2b1 was strengthened under hypoxia (Fig. 4B). RNA-FISH/IF showed that *Snhg18* and Hnrnpa2b1 could colocalize in mPASMCs (Fig. 4C).

As shown in Fig. 4D and E, increased Hnrnpa2b1 expression in HPH models was detected by Western blot assays and subsequently confirmed by immunofluorescence staining of pulmonary arteries. Moreover, qRT-PCR analysis and Western blot assays showed that Hnrnpa2b1 was increased under hypoxia, but knockdown of *Snhg18* had no effect on Hnrnpa2b1 expression in either mPASMCs or HPASMCs (Fig. 4F and G, Figure S4A and S4B), suggesting that *Snhg18* may post-transcriptionally regulate downstream targets through its interaction with Hnrnpa2b1.

### *Snhg18* binds to Hnrnpa2b1 to enhance *Eno3* mRNA stability

**Fig. 5:**
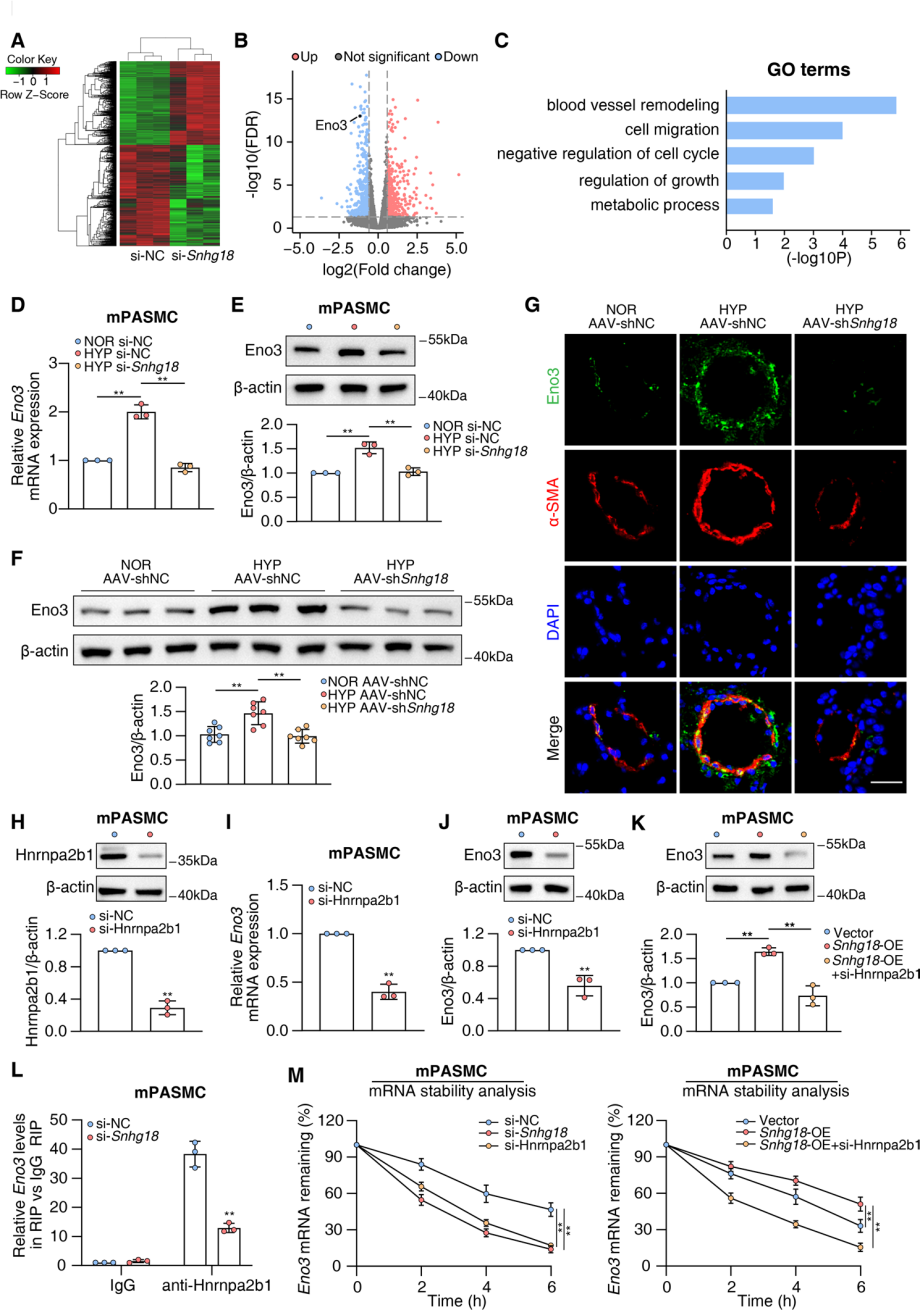
*Snhg18* promotes Eno3 expression by enhancing its mRNA stability via interaction with Hnrnpa2b1.** A** The heatmap of the differentially expressed genes in mPASMCs after knockdown of *Snhg18*. **B** The volcano plot of the differentially expressed genes in mPASMCs after knockdown of *Snhg18*. **C** Gene Ontology analysis of differentially expressed genes after inhibition of *Snhg18* in mPASMCs. **D** qRT-PCR analysis of *Eno3* expression in mPASMCs after indicated treatments. **E** Western blot analysis and quantification of Eno3 expression in mPASMCs after indicated treatments. **F** Western blot analysis and quantification of Eno3 expression in mouse lung tissues (*n* = 7). **G** Representative images of IF staining of PAs for Eno3 (green), α-SMA (red), and nuclei (blue). Scale bar 20 μm. **H** The efficiency of Hnrnpa2b1 knockdown was validated by Western blot analysis in mPASMCs. **I** qRT-PCR analysis of *Eno3* expression in mPASMCs after inhibition of Hnrnpa2b1. **J** Western blot analysis and quantification of Eno3 expression in mPASMCs after inhibition of Hnrnpa2b1. **K** Western blot analysis and quantification of Eno3 expression in mPASMCs after indicated treatments. **L** The RIP assays detected the binding between *Eno3* and Hnrnpa2b1 after knockdown of *Snhg18* in mPASMCs. **M** qRT-PCR analysis of *Eno3* expression at the indicated time after Actinomycin D treatment in mPASMCs. **D**-**F**, **K**, **M** The data were analyzed using one-way ANOVA with Dunnett’s multiple comparisons test, (**H-J**, **L**) The data were analyzed using two-tailed Student’s t-test, Data are presented as mean ± SD. **P < 0.01. NOR indicates normoxia; HYP, hypoxia; AAV, adeno-associated virus; DAPI, 4ʹ,6-diamidino-2-phenylindole, and OE indicates overexpression

To explore the underlying mechanism by which *Snhg18* functions in the pathogenesis of PH, we performed RNA sequencing after knockdown of *Snhg18* to identify its key downstream genes (Fig. 5A and B). Gene ontology (GO) analysis showed that the differentially expressed genes were enriched in terms related to blood vessel remodeling, regulation of growth, and metabolic process (Fig. 5C). Among the candidate genes, Eno3 was chosen for further exploration because of its role in cell proliferation and metabolism [41]. qRT-PCR analysis and Western blot assays showed that Eno3 expression was upregulated at both the mRNA and protein levels under hypoxia, and inhibition of *Snhg18* attenuated this effect in both mPASMCs and HPASMCs (Fig. 5D and E, Figure S5A and S5B). Additionally, we examined the expression of Eno3 in mouse models to validate our in vitro findings. As shown in Fig. 5F, knockdown of *Snhg18* inhibited hypoxia-induced Eno3 expression in lung tissues. Immunofluorescence staining of pulmonary arteries further confirmed the inhibitory role of *Snhg18* in regulating Eno3 expression in mPASMCs (Fig. 5G). Furthermore, after knockdown of Hnrnpa2b1 in mPASMCs and HPASMCs (Fig. 5H and Figure S5C), Eno3 expression was reduced at both the mRNA and protein levels (Fig. 5I and J, Figure S5D and S5E), which was consistent with the effect of *Snhg18* knockdown. Western blot analysis indicated that overexpression of *Snhg18* in mPASMCs and HPASMCs induced upregulation of Eno3 protein levels, and inhibition of Hnrnpa2b1 abolished the effect (Fig. 5K and Figure S5F-S5H). To explore whether *Snhg18* regulates the binding of Hnrnpa2b1 to *Eno3*, we performed RIP assays and found that inhibition of *Snhg18* significantly attenuated the interaction between Hnrnpa2b1 and *Eno3* (Fig. 5L), confirming the indispensable role of *Snhg18* in their binding process. To further explore whether *Snhg18* regulates *Eno3* mRNA stability through Hnrnpa2b1, we determined the remaining *Eno3* mRNA after actinomycin D treatment. qRT-PCR analysis showed a reduced half-life of *Eno3* mRNA after knockdown of *Snhg18* or Hnrnpa2b1, and the stabilizing effect induced by overexpression of *Snhg18* was abolished by Hnrnpa2b1 inhibition (Fig. 5M).

### Eno3 regulation depends on m6A modification

**Fig. 6:**
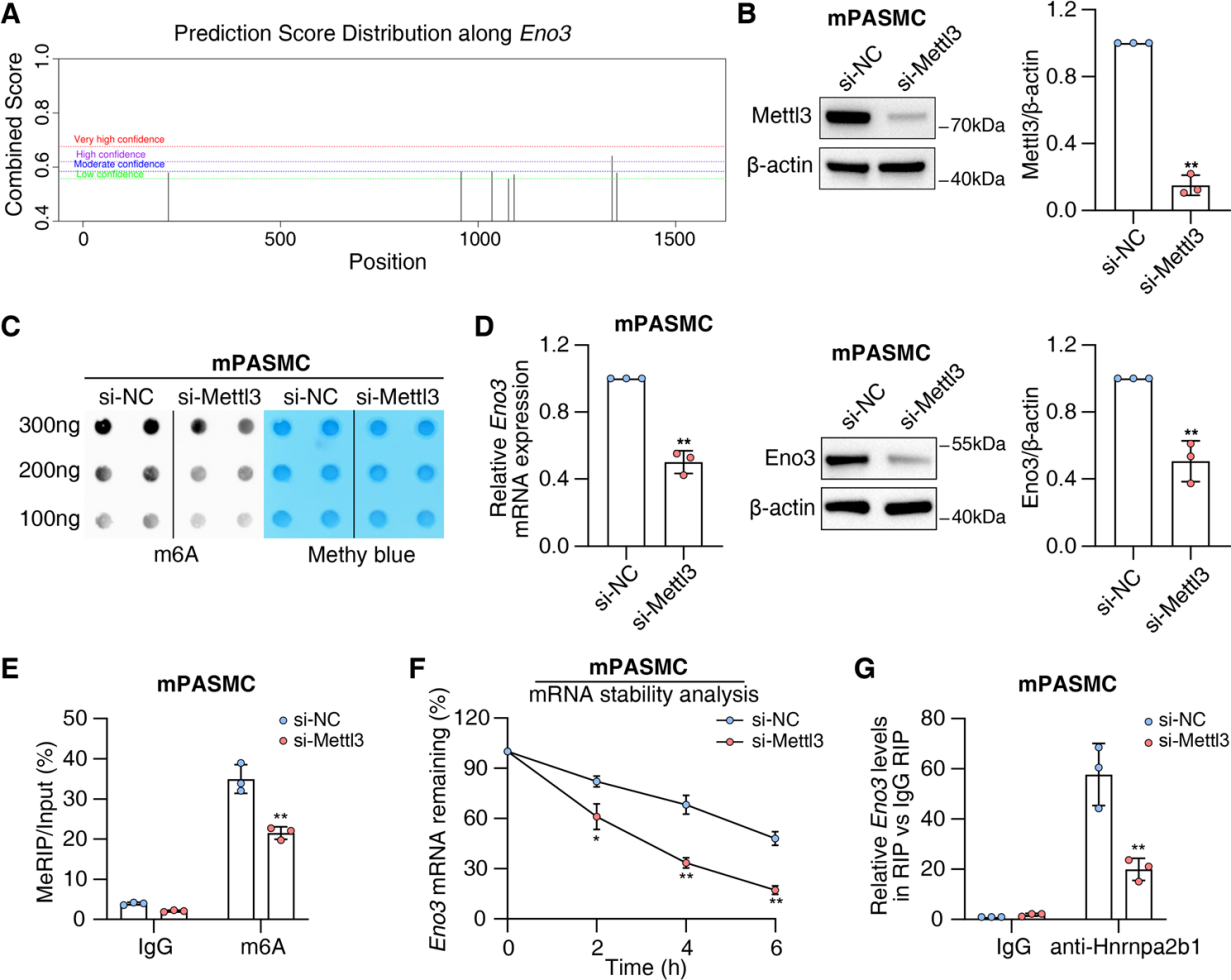
The regulation of Eno3 depends on m6A modification. **A** The specific m6A peak distribution of *Eno3* predicted by SRAMP. **B** The efficiency of knockdown of Mettl3 was validated by Western blot analysis in mPASMCs. **C** m6A dot blot assays detected overall m6A modification level after knockdown of Mettl3 in mPASMCs. **D** qRT-PCR and Western blot analysis of Eno3 expression after knockdown of Mettl3 in mPASMCs. **E** The MeRIP-qPCR assays detected the m6A modification level of *Eno3* after knockdown of Mettl3 in mPASMCs. **F** qRT-PCR analysis of *Eno3* mRNA expression at the indicated time after Actinomycin D treatment in mPASMCs. **G** The RIP assays detected the interaction between *Eno3* and Hnrnpa2b1 after knockdown of Mettl3 in mPASMCs. **B**, **D**, **E**-**G** The data were analyzed using two-tailed Student’s t-test. Data are presented as mean ± SD. *P < 0.05, **P < 0.01

Hnrnpa2b1 is known as an m6A reader that regulates m6A-modified genes, so we wondered whether *Snhg18* mediates Hnrnpa2b1-dependent regulation of Eno3 in an m6A-dependent manner. We predicted the m6A-modified region of *Eno3* using SRAMP (http://www.cuilab.cn/sramp) [42], and identified a high-confidence m6A modification site (Fig. 6A). Methyltransferase 3 (Mettl3) is a major RNA methyltransferase gene. We first knocked down its expression (Fig. 6B), and found a decrease in global m6A modification levels by m6A dot blot assays (Fig. 6C). Moreover, qRT-PCR analysis and Western blot assays demonstrated a reduction in Eno3 expression after knockdown of Mettl3 in both mPASMCs and HPASMCs (Fig. 6D and Figure S6A-S6C). Next, we conducted MeRIP-qPCR assays and found that m6A methylation of *Eno3* was reduced by knockdown of Mettl3, confirming that *Eno3* was modified by m6A (Fig. 6E). In addition, qRT-PCR analysis showed a decreased half-life of *Eno3* after inhibition of Mettl3 (Fig. 6F). Meanwhile, the interaction between Hnrnpa2b1 and *Eno3* was significantly reduced after Mettl3 knockdown (Fig. 6G).

### *Snhg18*/Hnrnpa2b1/Eno3 axis enhances glycolysis to promote HPH

**Fig. 7:**
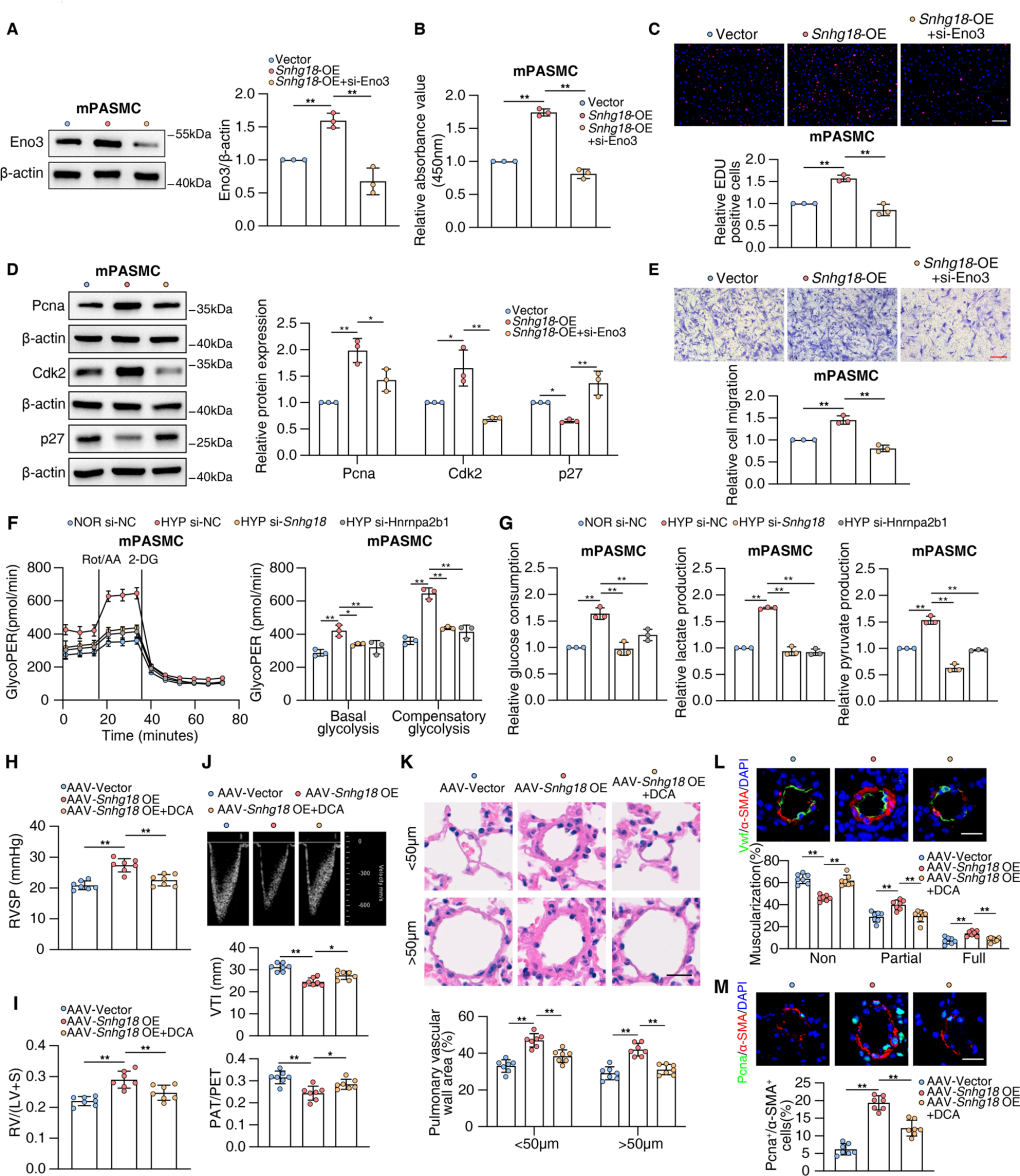
*Snhg18* promotes PH progression by regulating glycolysis.** A** Western blot analysis and quantification of Eno3 expression in mPASMCs. **B** CCK8 assays determined the proliferation of mPASMCs after indicated treatments. **C** EDU assays determined the proliferation of mPASMCs after indicated treatments. Scale bar 200 μm. **D** Western blot analysis and quantification of Pcna, Cdk2, and p27 expression in mPASMCs. (**E)** Transwell assays determined the migratory ability of mPASMCs after indicated treatments. Scale bar 200 μm. **F** ECAR assays evaluated the glycolytic profile of mPASMCs after indicated treatments, with quantification of basal and compensatory glycolysis. **G** The alteration of glucose consumption, lactate production, and pyruvate production in mPASMCs after indicated treatments. **H-J** RVSP (**H**), the ratio of right ventricular (RV) to left ventricular (LV) plus septum (S) (**I**), the velocity time integral (VTI), and the ratio of pulmonary artery acceleration time to ejection time (PAT/PET) (**J**) in PH model mice (*n* = 7). **K** Representative images of H&E staining of pulmonary arteries (PAs) and the quantification of vascular wall area index. Scale bar 20 μm. **L** Representative images of IF staining of PAs for Vwf (green), α-SMA (red), and nuclei (blue). The proportion of non, partially, or fully muscularized PAs was analyzed. Scale bar 20 μm. **M** Representative images of IF staining of PAs for Pcna (green), α-SMA (red) and nuclei (blue) and the quantification of Pcna^+^ cells in the lung section. Scale bar 20 μm. **A-M** The data were analyzed using one-way ANOVA with Dunnett’s multiple comparisons test. Data are presented as mean ± SD. *P < 0.05, **P < 0.01. OE indicates overexpression; NOR, normoxia; HYP, hypoxia; AAV indicates adeno-associated virus; RVSP, right ventricular systolic pressure; RV/(LV + S), right ventricular/ (left ventricle + septum); VTI, velocity time integral; PAT/PET, pulmonary artery acceleration time/pulmonary ejection time; and DAPI, 4ʹ,6-diamidino-2-phenylindole

To explore the role of the *Snhg18*/Hnrnpa2b1/Eno3 axis in PASMCs during HPH progression, we knocked down Eno3 in *Snhg18*-overexpressing mPASMCs and HPASMCs (Fig. 7A and Figure S7A). CCK8 assays (Fig. 7B and Figure S7B) and EDU assays (Fig. 7C and Figure S7C) demonstrated that overexpression of *Snhg18* accelerated PASMC proliferation, which was attenuated by knockdown of Eno3. In addition, Western blot assays showed that elevation of *Snhg18* significantly upregulated the expression of Pcna and Cdk2 and repressed p27. Knockdown of Eno3 could reverse these changes in both mPASMCs and HPASMCs (Fig. 7D and Figure S7D). Moreover, the augmented migratory ability of mPASMCs and HPASMCs induced by *Snhg18* overexpression was repressed by inhibition of Eno3 (Fig. 7E and Figure S7E).

As a vital glycolysis-related enzyme, Eno3 enhances glucose uptake and promotes glycolytic processes [41]. Extracellular acidification rate (ECAR) assays demonstrated that knockdown of *Snhg18* or Hnrnpa2b1 reduced the glycolytic capacity of mPASMCs activated under hypoxia, with decreased basal glycolysis and compensatory glycolytic capacity (Fig. 7F). Moreover, we found that hypoxia-induced glucose consumption, lactate production, and pyruvate production were decreased in *Snhg18* or Hnrnpa2b1 knockdown mPASMCs (Fig. 7G). In addition, previous studies have shown that the HIF signaling plays an important role in hypoxia-induced metabolic reprogramming [43, 44]. We further investigated the relationship between Hif-1α and *Snhg18*. We repressed Hif-1α expression in mPASMCs and HPASMCs, and then measured the expression of *Snhg18*. We found knockdown of Hif-1α did not lead to a significant change in *Snhg18* expression in mPASMCs and HPASMCs (Figure S8A and S8B). Moreover, *Snhg18* knockdown did not alter Hif-1α expression in mPASMCs and HPASMCs (Figure S8C), suggesting that *Snhg18* may regulate glycolysis independently of the HIF signaling.

To further confirm that *Snhg18* promotes PH development by regulating glycolysis in vivo, we performed rescue experiments. After overexpression of *Snhg18* (Figure S9A and S9B), the mice were treated simultaneously with dichloroacetate (DCA), a well-established glycolysis inhibitor that has been demonstrated to suppress PH progression [45, 46]. As shown in Fig. 7H and I, RVSP and the ratio of RV to left ventricle plus septum were elevated after overexpression of *Snhg18*, and treatment with DCA could reverse this elevation. Echocardiography showed that the reduced VTI and PAT/PET caused by *Snhg18* overexpression could be ameliorated by the use of DCA (Fig. 7J). Besides, DCA treatment reduced the wall thickness and muscularization of vessels in *Snhg18*-overexpressing mice (Fig. 7K and L). Immunofluorescence staining for Pcna showed fewer proliferating mPASMCs in the pulmonary arteries after DCA treatment in *Snhg18*-overexpressing mice (Fig. 7M).

## Discussion

Pulmonary vascular remodeling is a major pathological feature of PH, and aggressive proliferation of PASMCs is a crucial contributing factor [47]. Among the complex mechanisms underlying PASMC hyperproliferation, lncRNA regulation has garnered increasing attention. For instance, the lncRNA *Hoxaas3* accelerates the cell cycle by regulating cyclin A, cyclin D, and cyclin E to induce HPH [48]. Emerging evidence indicates the important role of the lncRNA small nucleolar RNA host gene (Snhg) family in modulating PH progression. *SNHG5* forms a positive feedback loop with EPAS1 to promote vascular remodeling by activating the transactivation of ICAM1 [49]. In this study, we demonstrated the aberrant expression of the lncRNA *Snhg18* in both HPH model tissues and PASMCs treated under hypoxia. In addition, our in vitro and in vivo data confirmed that knockdown of *Snhg18* inhibited PASMC proliferation and pulmonary vascular remodeling under hypoxia.

Similar to protein-coding genes, the expression of lncRNAs is also controlled through genomic and transcriptional regulation. At the genomic level, copy number variations have been reported in the predominant genes related to the development of PH, including GDF2 [31], BMPR2 [33], and TBX4 [50]. We found that *Snhg18* expression was upregulated in PASMCs and PH model tissues with CNA, and the *Snhg18* copy number was increased in the plasma of PH patients. Given that hypoxia is a systemic condition, SNHG18 copy number amplification may also occur in other organs, and amplification may also occur in smooth muscle cells or endothelial cells in other tissues, which warrants further investigation in future studies. We also revealed that the transcription factor Sp1 facilitated *Snhg18* transcription by binding to its promoter, thus increasing the expression of *Snhg18* under hypoxia. Previous studies have demonstrated the pivotal role of Sp1 in PH progression [37, 51]. We uncovered the activation mechanism of *Snhg18* at both the genomic and transcriptional levels in the pathogenesis of PH.

LncRNAs regulate downstream genes by binding to RNA-binding proteins (RBPs) [52]. In our study, the interaction between lncRNA *Snhg18* and the RBP Hnrnpa2b1 was identified. While knockdown of *Snhg18* had no influence on Hnrnpa2b1 expression, it reduced the binding of Hnrnpa2b1 to *Eno3* mRNA, thereby impaired PASMC proliferation. Ruffenach et al. demonstrated increased expression of Hnrnpa2b1 in pulmonary arterial hypertension, where it regulated the cell cycle by binding to mRNAs [53]. In addition, Hnrnpa2b1 has been recognized as an m6A reader protein. As a prevalent form of RNA epigenetic modification, aberrant m6A modification has been extensively studied in PH pathogenesis. For example, recognition of m6A-modified *MAGED1* mRNA by the m6A reader YTHDF1 promotes translation, thereby facilitating PH progression [17]. Our study demonstrated that the *Snhg18*/Hnrnpa2b1 axis can promote PH progression by regulating m6A methylation.

Emerging evidence suggests that Hnrnpa2b1 regulates mRNA stability by recognizing m6A sites on its target RNA transcripts [39, 54]. Given that Mettl3 is the key methyltransferase catalyzing m6A modification, we knocked down Mettl3 to reduce global m6A methylation in PASMCs. Our results demonstrated that suppression of m6A modification impaired the interaction between Hnrnpa2b1 and *Eno3* mRNA, consequently destabilizing *Eno3* transcripts and reducing its expression, which was dependent on *Snhg18*. Thus, m6A modification of *Eno3* was essential for both its stabilization and its interaction with Hnrnpa2b1. We provided evidence that lncRNAs can control downstream gene expression via an m6A-dependent mechanism in PASMCs.

Glucose is a critical energy source for cellular metabolism. Growing evidence suggests that metabolic remodeling plays a pivotal role in PH pathogenesis. As an important mode of glucose metabolism, glycolysis is capable of supplying energy for the rapid proliferation of cells, converting glucose into pyruvate, and ultimately lactate [19]. The dysregulated expression of glycolysis-related enzymes has been reported to participate in the progression of PH [55–57]. Eno3 functions as a glycolytic metalloenzyme that catalyzes the reversible reaction of 2-phosphoglycerate to phosphoenolpyruvate, a vital step of glycolysis [58]. Eno3-mediated promotion of glycolysis in cell proliferation has been reported in cancer [59, 60], but there are scarce studies in PH. LncRNAs play an important role in various diseases by modulating glycolysis, including cancers [61], cerebral ischemia/reperfusion injury [62], and male infertility [63]. It has been demonstrated that the lncRNA *KMT2E-AS1* could enhance glycolysis in pulmonary artery endothelial cells [64]. However, the role of the crosstalk between lncRNAs and *ENO3* in PASMCs remains to be clarified. In our study, we observed a decreased glycolytic rate using ECAR assays after knockdown of *Snhg18/*Hnrnpa2b1 and confirmed reduced glucose consumption along with lactate and pyruvate production. We demonstrated that the lncRNA Snhg18/Hnrnpa2b1/Eno3 axis could regulate glycolysis in PASMCs.

## Conclusions

**Fig. 8:**
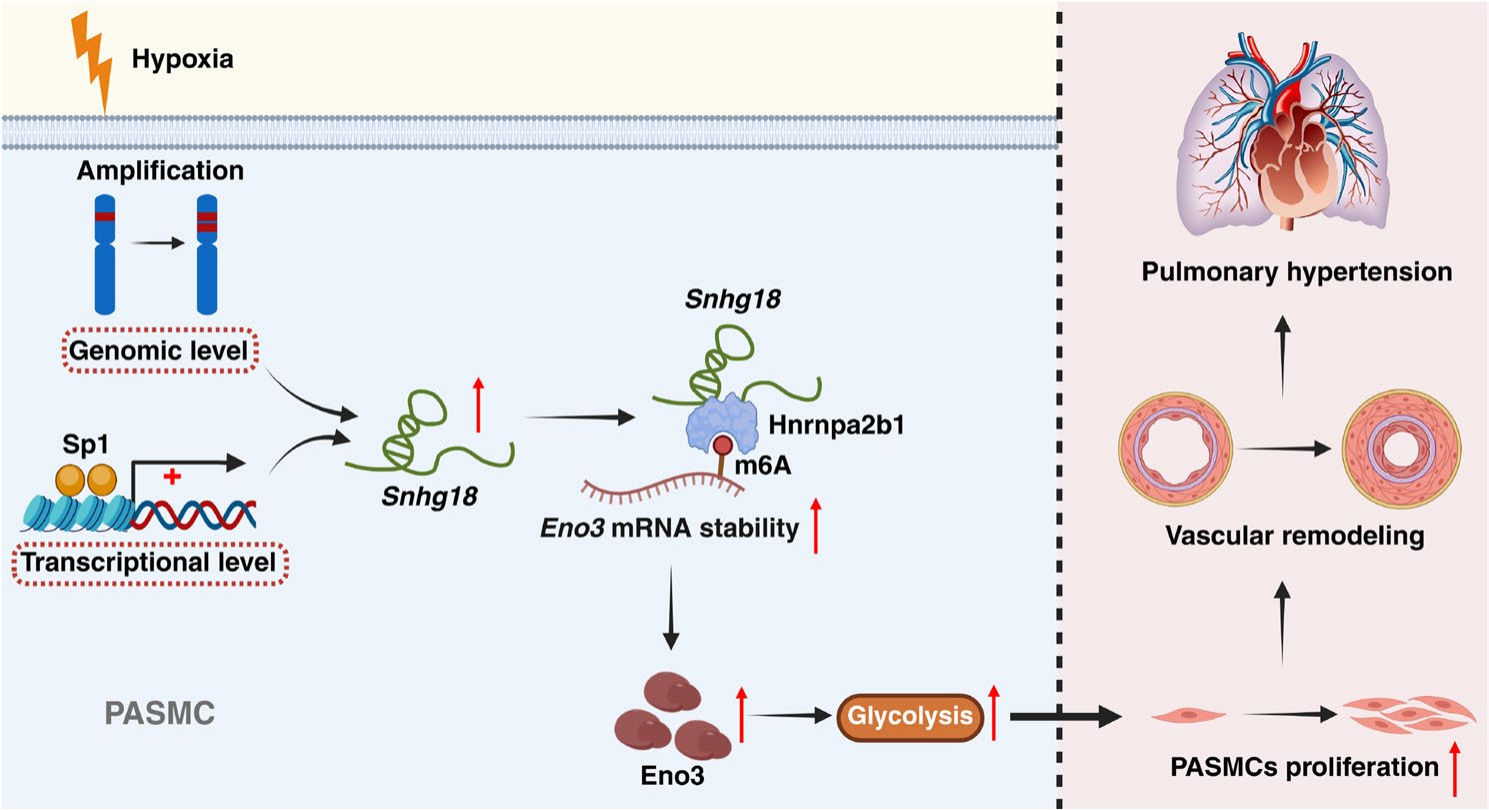
The schematic model of *Snhg18* in hypoxic pulmonary hypertension

In conclusion, we found that *Snhg18*, activated by CNA and Sp1, binds to Hnrnpa2b1 and subsequently facilitates glycolysis by enhancing *Eno3* mRNA stability through an m6A-dependent mechanism (Fig. 8). Our study provides new insights into the role of *Snhg18* in promoting PASMC proliferation and pulmonary vascular remodeling in HPH, suggesting that targeting *Snhg18* may be a promising therapeutic strategy for PH.

## Supplementary Information

Supplementary Material 1.

Supplementary Material 2.

## Data Availability

The mass spectrometry proteomics data has been deposited to the ProteomeXchange Consortium via the PRIDE partner repository with the dataset identifier PXD070529 (http://www.ebi.ac.uk/pride). The RNA-seq data has been deposited to the Gene Expression Omnibus (GEO) under accession number GSE305837 (https://www.ncbi.nlm.nih.gov/geo/query/acc.cgi?acc=GSE305837).

## Abbreviations

Snhg18: Small nucleolar RNA host gene 18
Eno3: Enolase 3
PH: Pulmonary hypertension
LncRNA: Long non-coding RNA
PASMC: Pulmonary artery smooth muscle cell
Sp1: Sp1 transcription factor
m6A: N6-methyladenosine
Hnrnpa2b1: Heterogeneous nuclear ribonucleoprotein A2/B1
AAV9: Adeno-associated virus 9
shRNA: Short-hairpin RNA
RVSP: Right ventricular systolic pressure
RVH: Right ventricular hypertrophy
mPASMC: Mouse pulmonary artery smooth muscle cell
HPASMC: Human pulmonary artery smooth muscle cell
siRNA: Small interfering RNA
ECAR: Extracellular acidification rate
HPH: Hypoxia-induced pulmonary hypertension
CNA: Copy number amplification
Mettl3: Methyltransferase 3
