# Supplementary material for: Snhg18 promotes hypoxic pulmonary hypertension by enhancing glycolysis
Source: Respir Res. 2026 May 18;27:289. doi: 10.1186/s12931-026-03707-1 (PMC13390410; doi:10.1186/s12931-026-03707-1)

Full unedited gel for Figure 2D

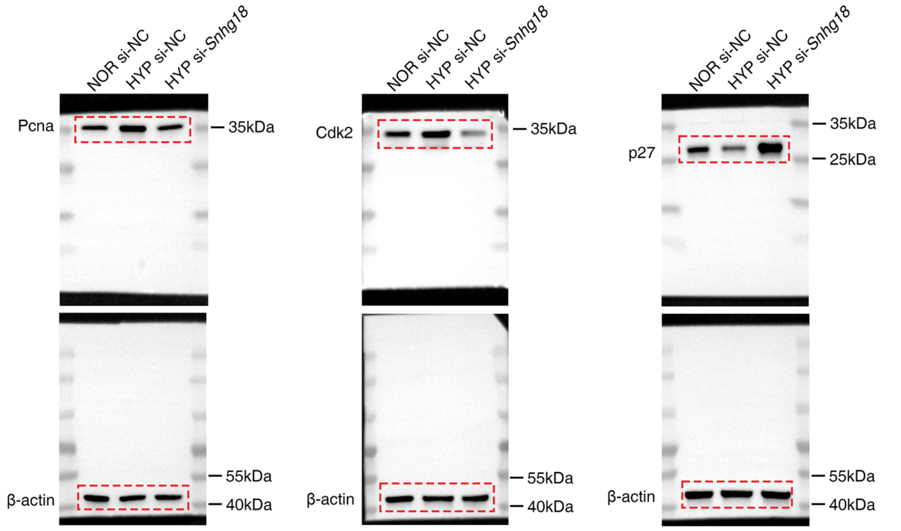

Full unedited gel for Figure 3E

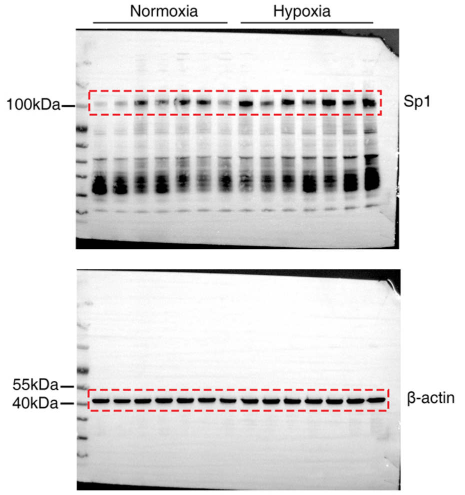

Full unedited gel for Figure 3G

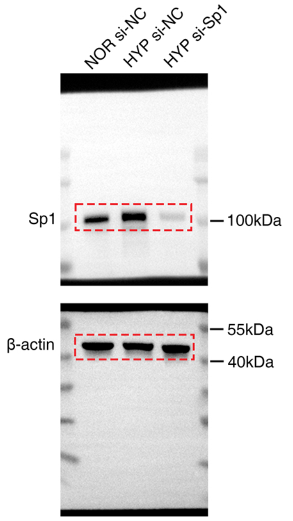

Full unedited gel for Figure 3I

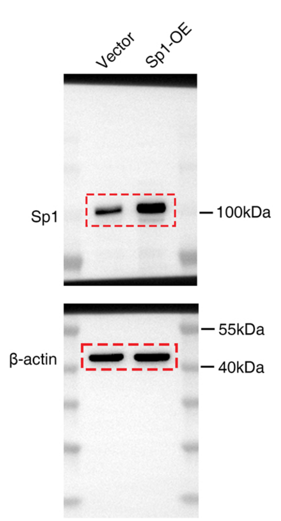

Full unedited gel for Figure 4A

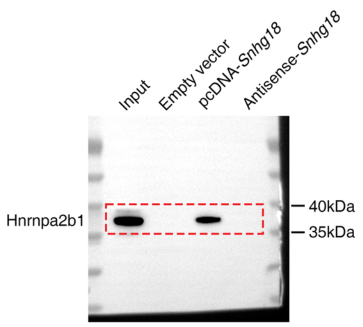

Full unedited gel for Figure 4D

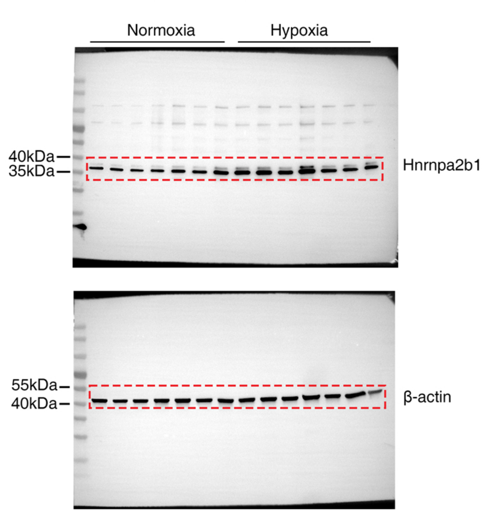

Full unedited gel for Figure 4G

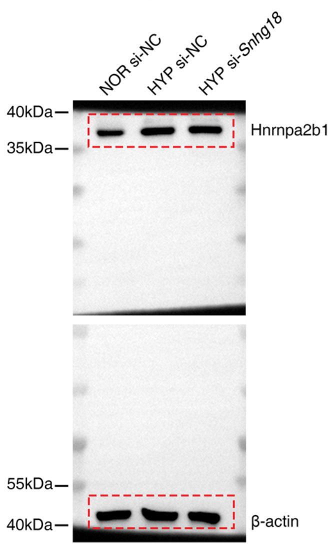

Full unedited gel for Figure 5E

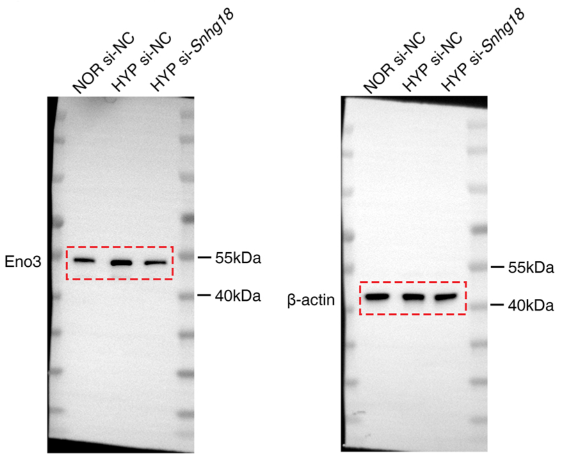

Full unedited gel for Figure 5F

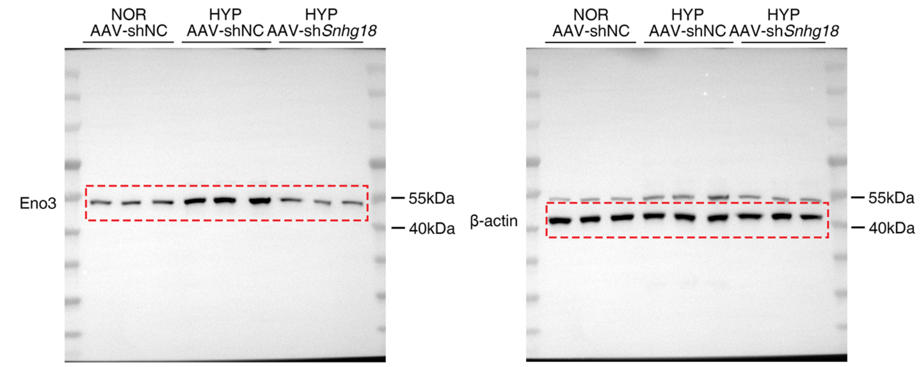

Full unedited gel for Figure 5H

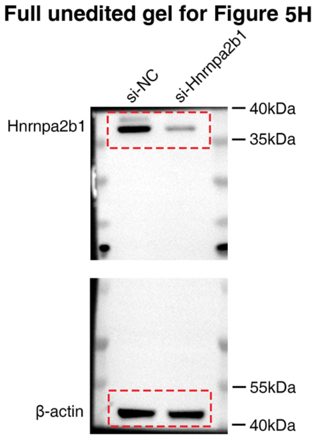

Full unedited gel for Figure 5J

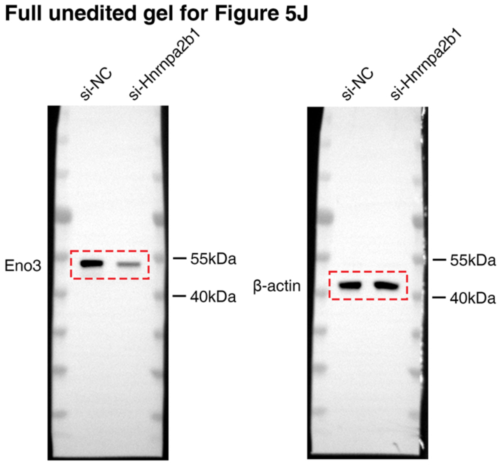

Full unedited gel for Figure 5K

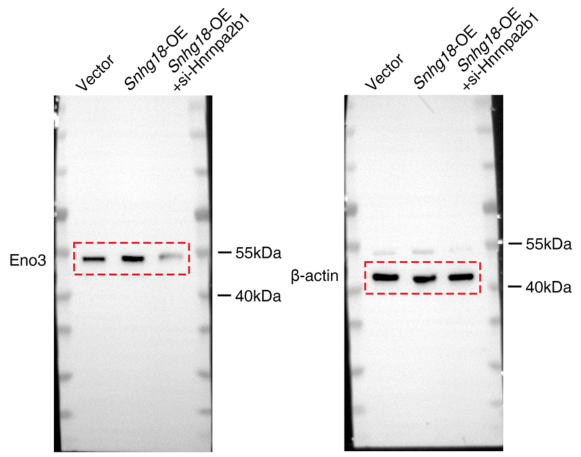

Full unedited gel for Figure 6B

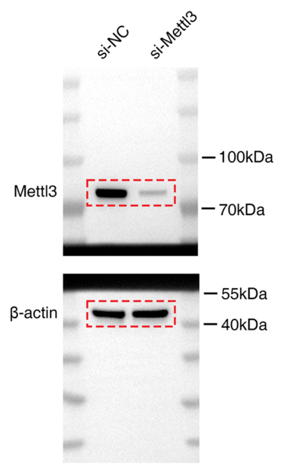

Full unedited gel for Figure 6D

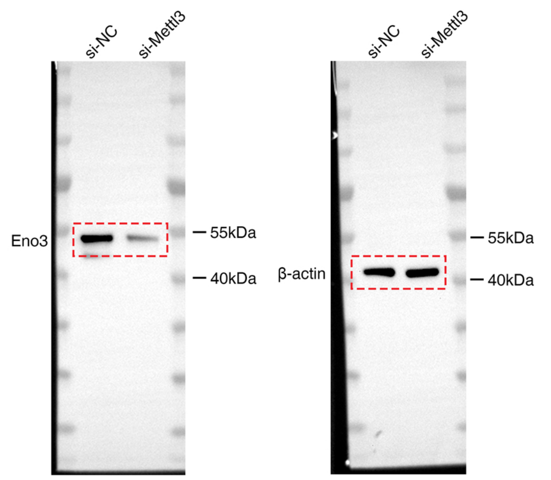

Full unedited gel for Figure 7A

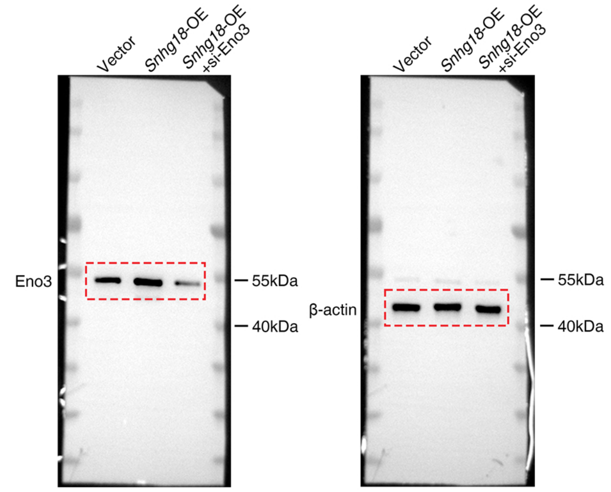

Full unedited gel for Figure 7D

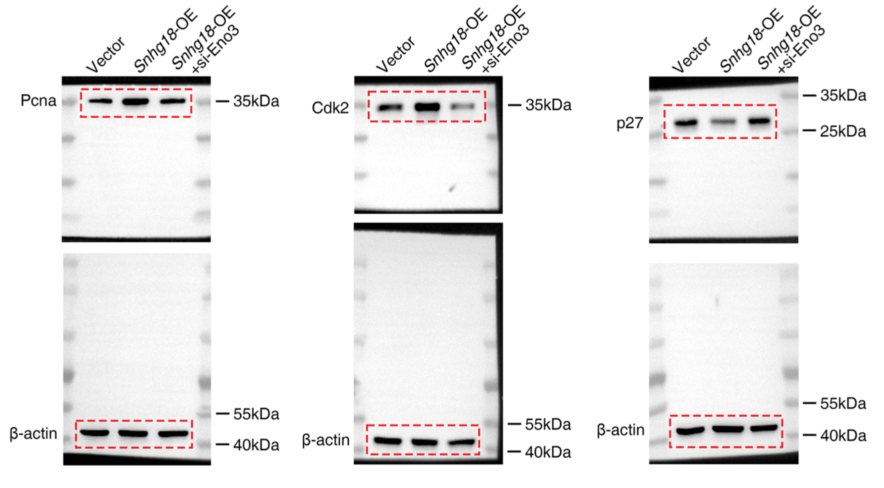

Full unedited gel for Figure S1D

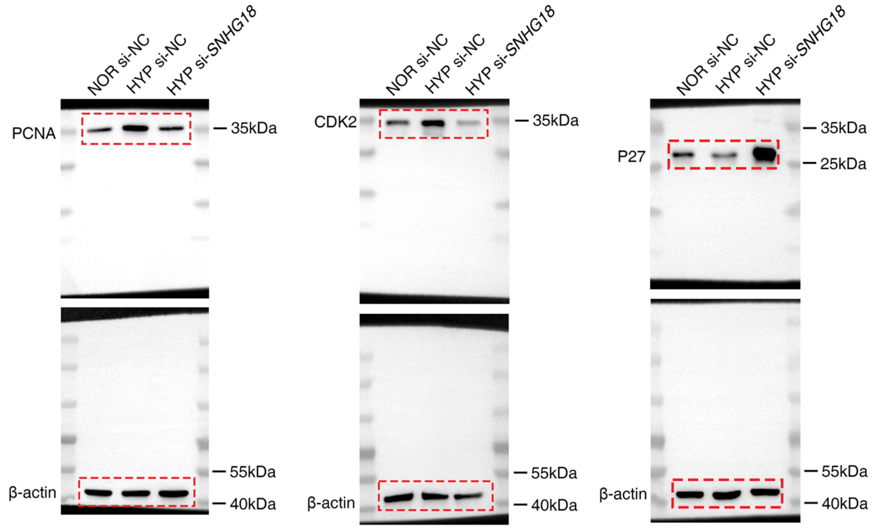

Full unedited gel for Figure S3A

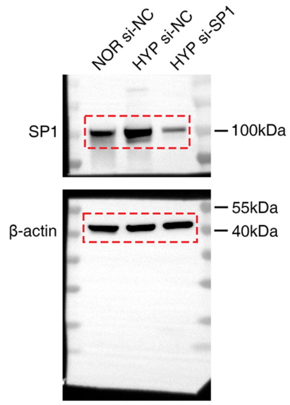

Full unedited gel for Figure S3C

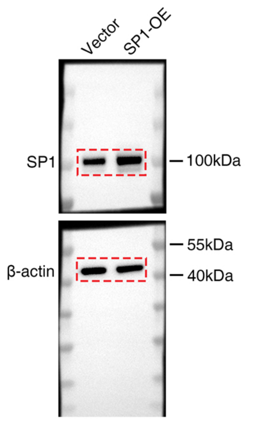

Full unedited gel for Figure S4B

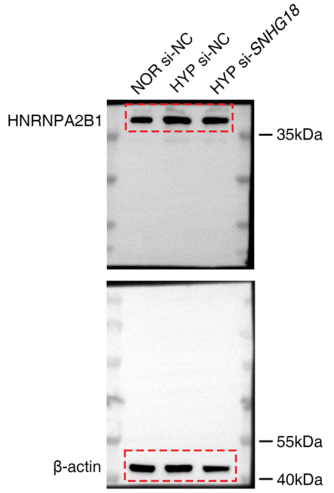

Full unedited gel for Figure S5B

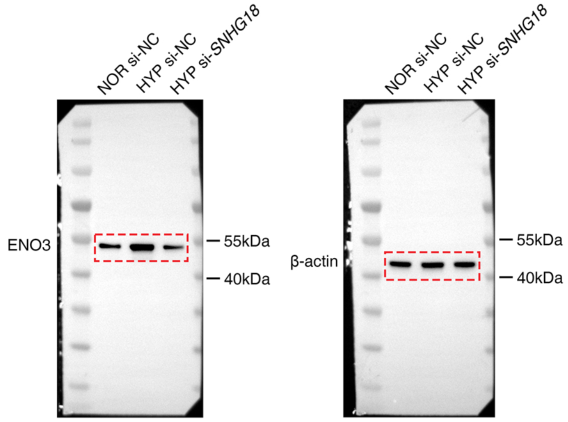

Full unedited gel for Figure S5C

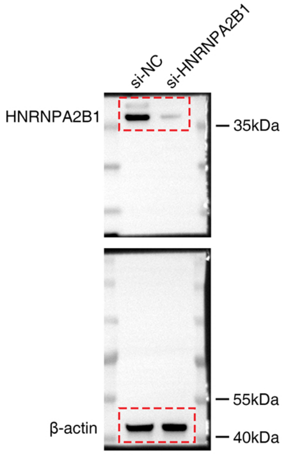

Full unedited gel for Figure S5E

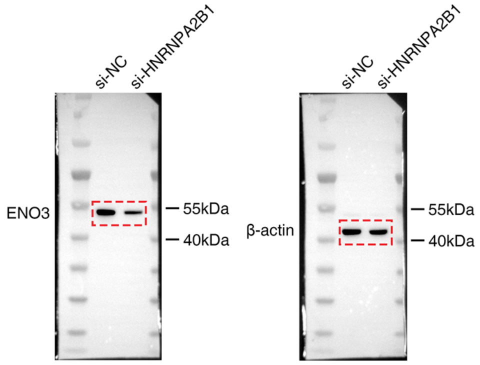

Full unedited gel for Figure S5H

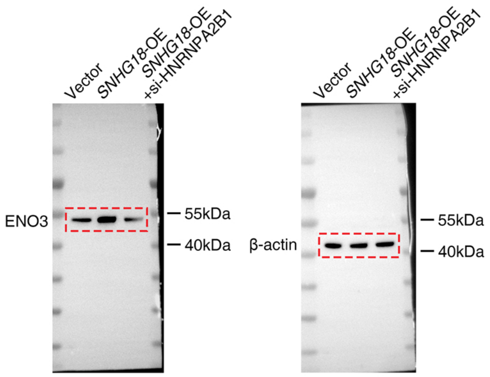

Full unedited gel for Figure S6A

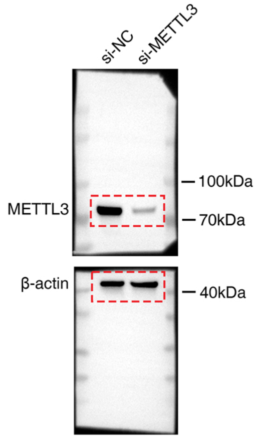

Full unedited gel for Figure S6C

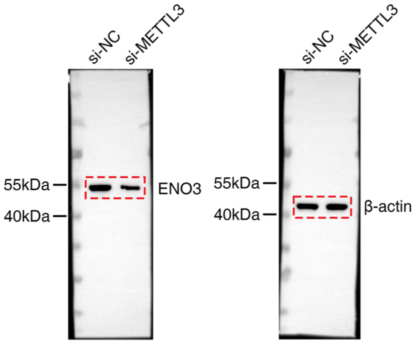

Full unedited gel for Figure S7A

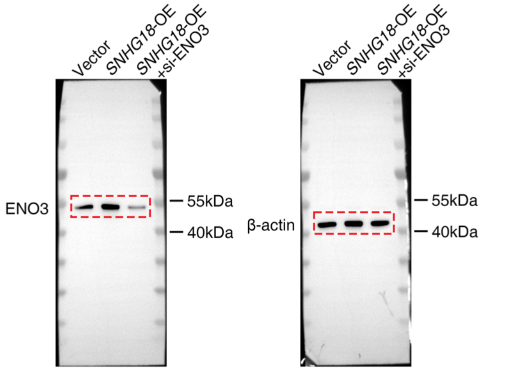

Full unedited gel for Figure S7D

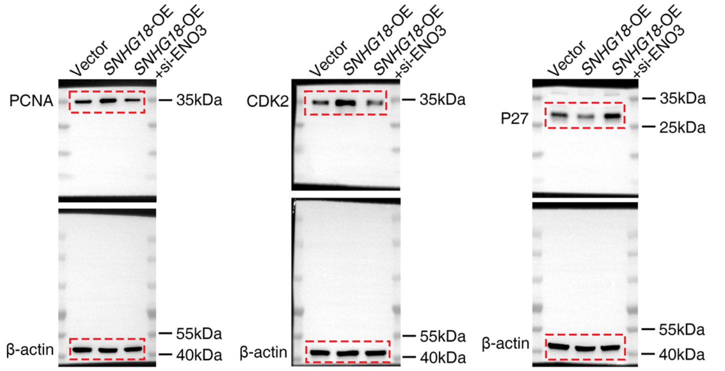

Full unedited gel for Figure S8A

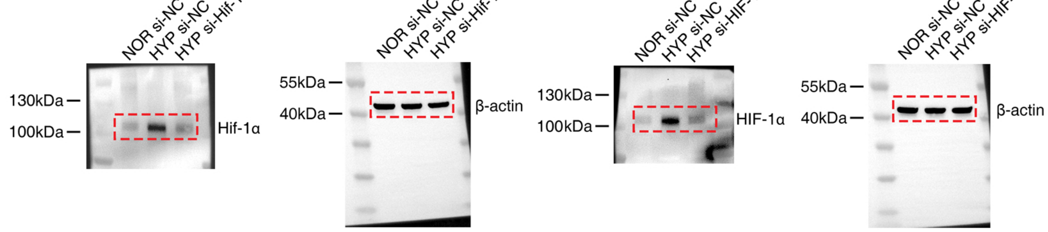

Full unedited gel for Figure S8C

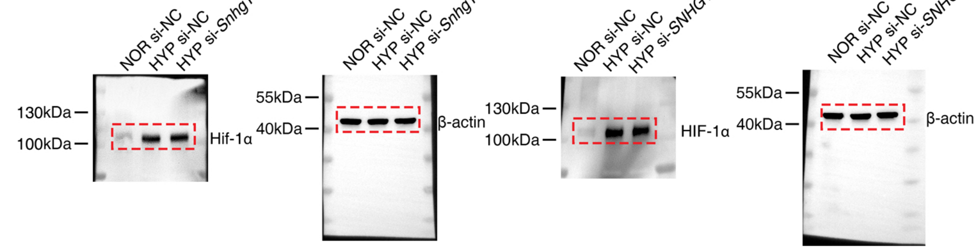

Supplement: Supplementary file 1 — Supplementary Material 1. [file 12931_2026_3707_MOESM1_ESM.pdf]
