# Supplementary material for: Snhg18 promotes hypoxic pulmonary hypertension by enhancing glycolysis
Source: Respir Res. 2026 May 18;27:289. doi: 10.1186/s12931-026-03707-1 (PMC13390410; doi:10.1186/s12931-026-03707-1)
Supplement: Supplementary file 2 — Supplementary Material 2. [file 12931_2026_3707_MOESM2_ESM.pdf]

SUPPLEMENTAL INFORMATION

Snhg18 promotes hypoxic pulmonary hypertension by enhancing glycolysis

Tianyu Qu[1#], Kai Ma[1#], Qiang Du[2], Chun Zhang[3], Ziling Shen[1], Enze Wang[1], Zhixuan Chen[1], Pingsheng Chen[4], Yingying Liu[1*], Ruifeng Zhang[1*]

Authors' Affiliations:

[1]Department of Respiratory Medicine, Zhongda Hospital, School of Medicine, Southeast University, 87 Dingjiaqiao, Nanjing 210009, Jiangsu, China;

[2]Department of Respiratory and Critical Care Medicine, Suzhou Municipal Hospital, Gusu School, The Affiliated Suzhou Hospital of Nanjing Medical University, Nanjing Medical University, 242 Guangji Road, Soochow, 215000, Jiangsu, P.R. China;

[3]Department of Tuberculosis, The Second Hospital of Nanjing, Nanjing University of Chinese Medicine, Nanjing, 210009, China;

[4]Department of Pathology, School of Medicine, Southeast University, Nanjing, China.

[#] These authors contributed equally to this work.

***Correspondence author:**

Ruifeng Zhang

Department of Respiratory Medicine, Zhongda Hospital, School of Medicine, Southeast University, 87 Dingjiaqiao, Nanjing 210009, Jiangsu, China;

Tel: +86 13851407854

E-mail: zrf1977313@yeah.net

Yingying Liu

Department of Respiratory Medicine, Zhongda Hospital, School of Medicine, Southeast University, 87 Dingjiaqiao, Nanjing 210009, Jiangsu, China.

Tel: +86 15751001245

E-mail: WXHwangliu@163.com

Running title: *Snhg18* promotes HPH by enhancing glycolysis.

Content:

Figure S1-S9; Table S1-S3

Figure S1

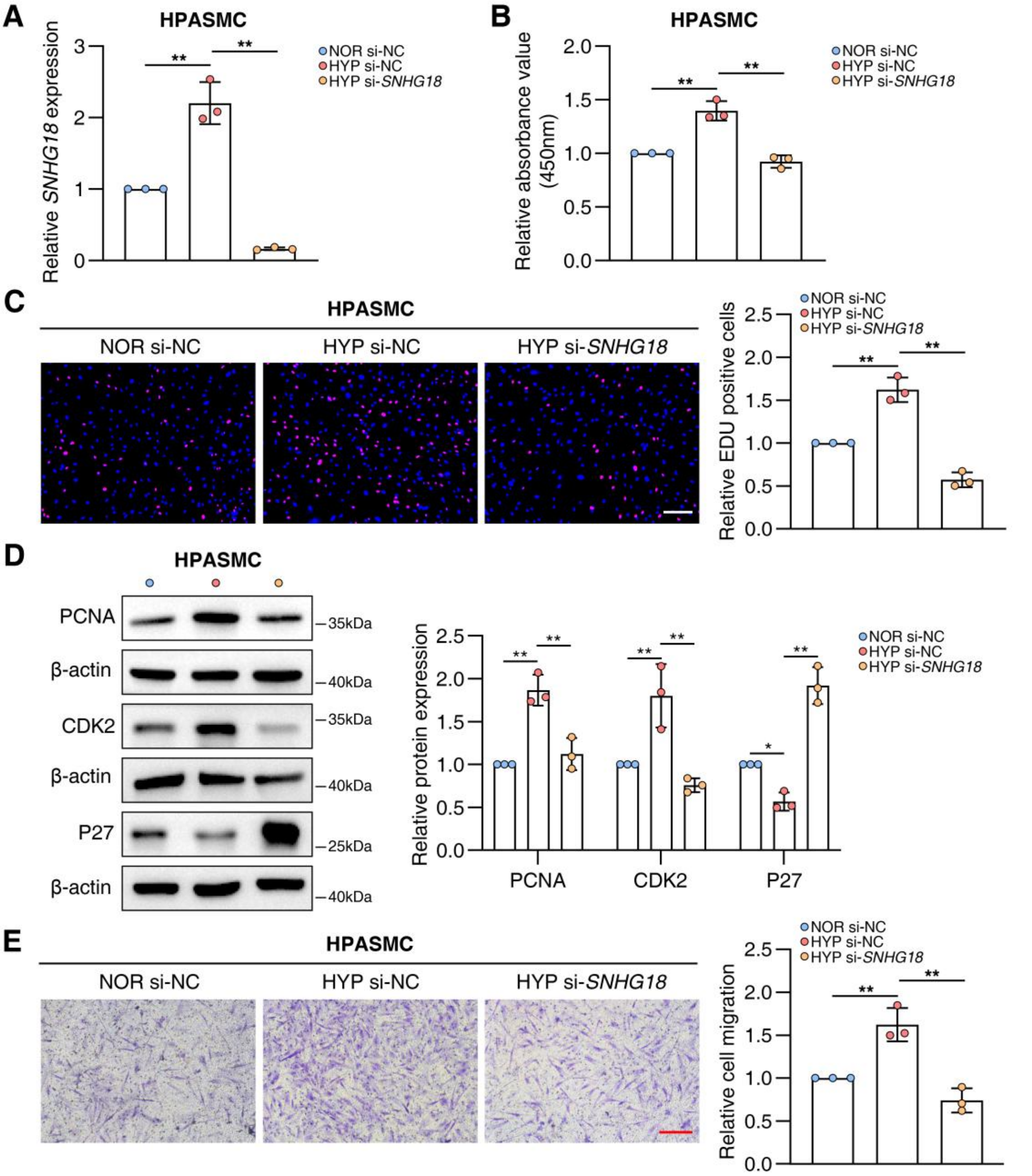

Figure S1 *SNHG18* promotes HPASMC proliferation in hypoxia. (**A**) qRT-PCR analysis of *SNHG18* expression in HPASMCs after indicated treatments. (**B**) CCK8 assays determined the proliferation of HPASMCs after indicated treatments. (**C**) EDU assays determined the proliferation of HPASMCs after indicated treatments. Scale bar 200 μm. (**D**) Western blot analysis and quantification of PCNA, CDK2, and P27 expression in HPASMCs. (**E**) Transwell assays determined the migratory ability of HPASMCs after indicated treatments. Scale bar 200 μm. (**A-E**) The data were analyzed

using one-way ANOVA with Dunnett's multiple comparisons test. Data are presented as mean ± SD. *$P < 0.05$, **$P < 0.01$. NOR indicates normoxia; and HYP, hypoxia.

Figure S2

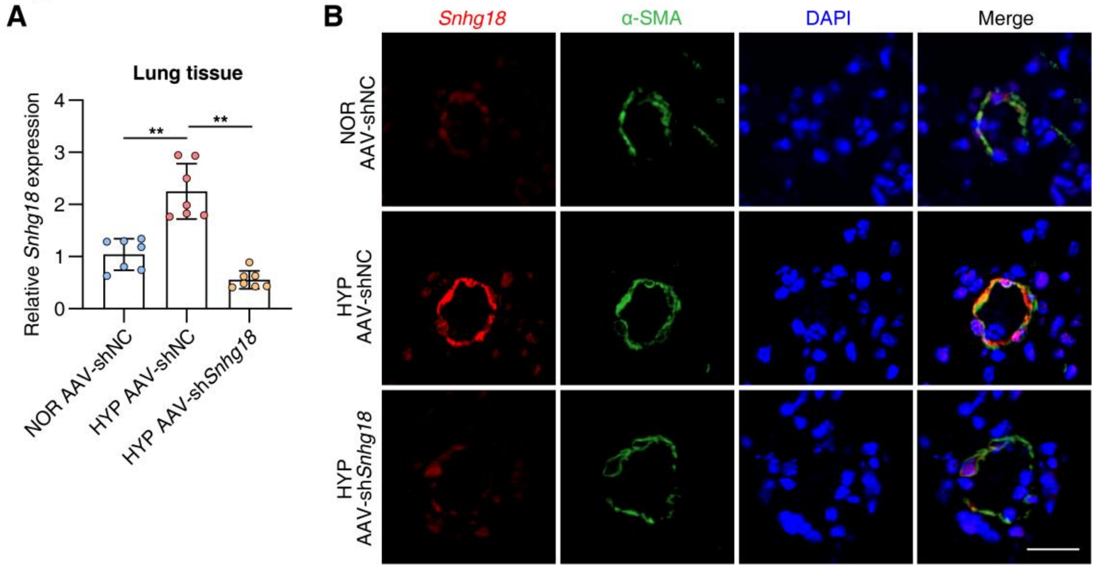

Figure S2 The knockdown efficiency of *Snhg18* in vivo. (**A**) qRT-PCR analysis of *Snhg18* expression in the mouse lung tissues (n=7). (**B**) RNA FISH-IF staining of PAs for *Snhg18* (red), α-SMA (green), and nuclei (blue). Scale bar 20 μm. (**A**) The data was analyzed using a one-way ANOVA with Dunnett's multiple comparisons test. Data are presented as mean ± SD. **$P < 0.01$. NOR indicates normoxia; HYP, hypoxia; AAV, adeno-associated virus; and DAPI, 4′,6-diamidino-2-phenylindole.

Figure S3

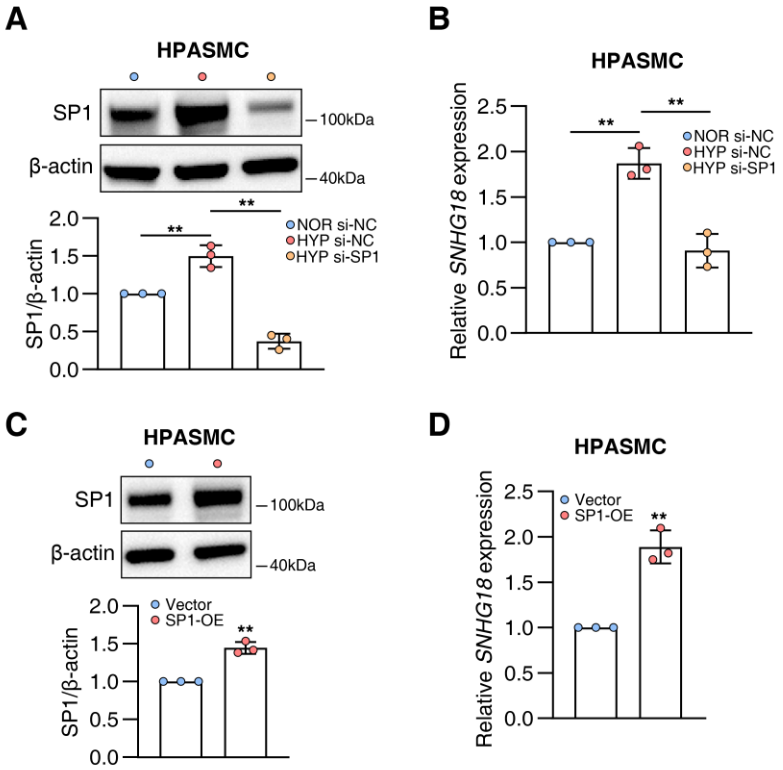

Figure S3 Regulation of *SNHG18* expression by SP1 in HPASMCs. (**A**) Western blot analysis and quantification of SP1 expression in HPASMCs. (**B**) qRT-PCR analysis of *SNHG18* expression in HPASMCs. (**C**) The efficiency of SP1 overexpression was validated by Western blot analysis in HPASMCs. (**D**) qRT-PCR analysis of *SNHG18* expression after SP1 overexpression in HPASMCs. (**A**, **B**) The data were analyzed using one-way ANOVA with Dunnett's multiple comparisons test. (**C**, **D**) The data were analyzed using two-tailed Student's t-test. Data are presented as mean ± SD. $^{**}P < 0.01$. NOR indicates normoxia; HYP, hypoxia; and OE, overexpression.

Figure S4

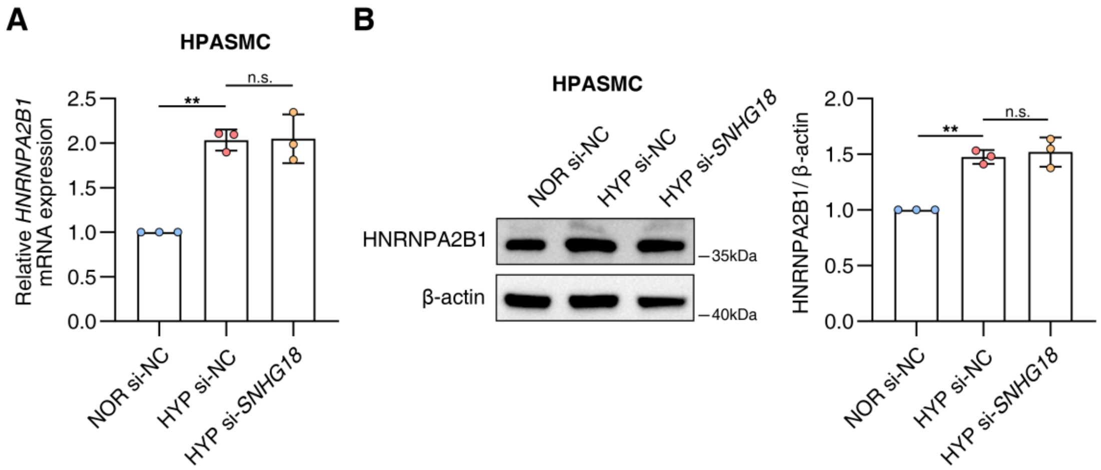

Figure S4 The expression of HNRNPA2B1 after *SNHG18* knockdown in HPASMCs. (**A**) qRT-PCR analysis of *HNRNPA2B1* expression in HPASMCs. (**B**) Western blot analysis and quantification of HNRNPA2B1 expression in HPASMCs. (**A**, **B**) The data were analyzed using one-way ANOVA with Dunnett's multiple comparisons test. Data are presented as mean ± SD. **P < 0.01. n.s., not significant. NOR indicates normoxia; and HYP, hypoxia.

Figure S5

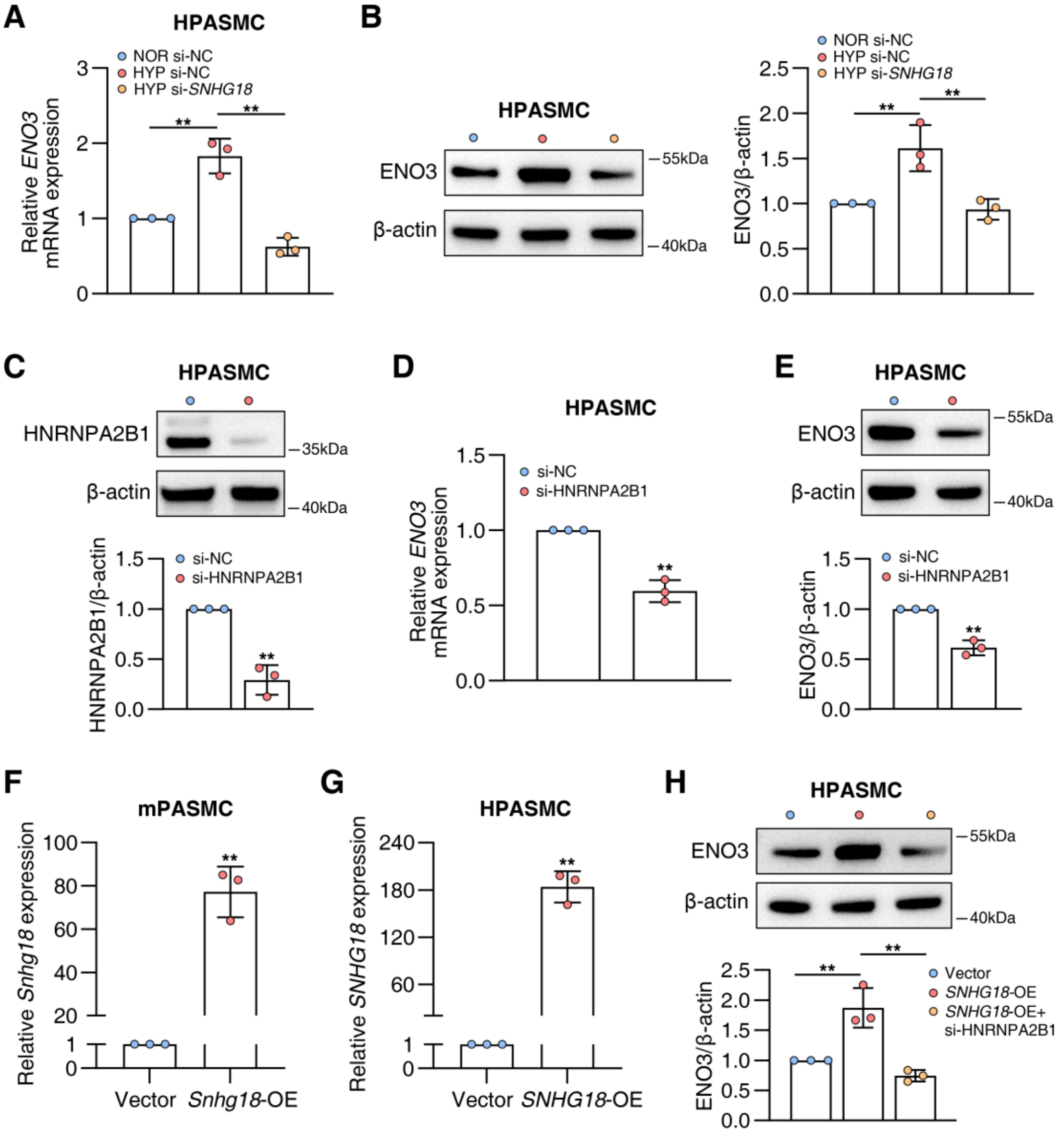

Figure S5 The regulation of *SNHG18*/HNRNPA2B1/ENO3 axis in HPASMCs. (**A**) qRT-PCR analysis of *ENO3* expression in HPASMCs after indicated treatments. (**B**) Western blot analysis and quantification of ENO3 expression in HPASMCs after indicated treatments. (**C**) The efficiency of knockdown of HNRNPA2B1 was validated by Western blot analysis in HPASMCs. (**D**) qRT-PCR analysis of *ENO3* expression after inhibition of HNRNPA2B1 in HPASMCs. (**E)** Western blot analysis and quantification of ENO3 expression after inhibition of HNRNPA2B1 in HPASMCs. (**F**) The efficiency of overexpression of *Snhg18* was validated by qRT-PCR analysis in

mPASMCs. (**G**) The efficiency of overexpression of *SNHG18* was validated by qRT-PCR analysis in HPASMCs. (**H**) Western blot analysis and quantification of ENO3 expression in HPASMCs after indicated treatments. (**A**, **B**, **H**) The data were analyzed using one-way ANOVA with Dunnett's multiple comparisons test. (**C-G**) The data were analyzed using two-tailed Student's t-test. Data are presented as mean ± SD. $^{**}P < 0.01$. NOR indicates normoxia; HYP, hypoxia; and OE, overexpression.

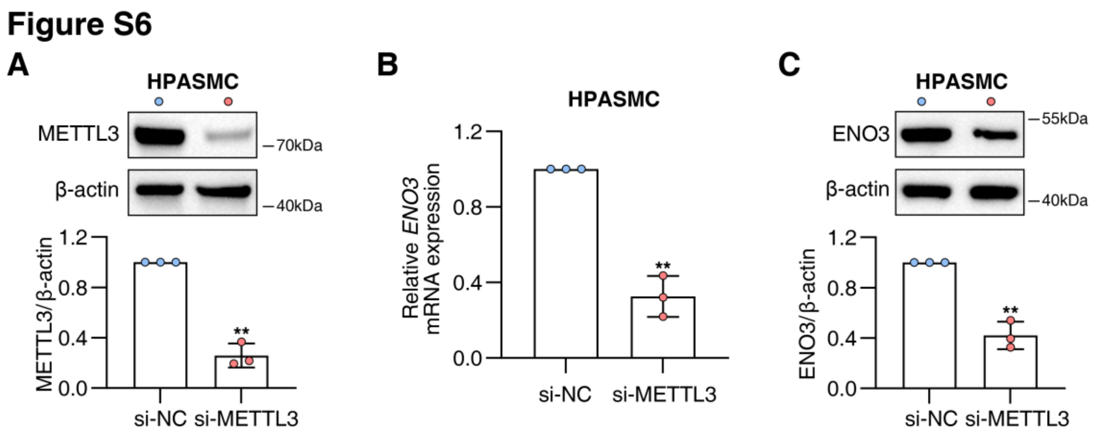

Figure S6 The expression of ENO3 after METTL3 knockdown in HPASMCs. (**A**) The efficiency of METTL3 knockdown was validated by Western blot analysis in HPASMCs. (**B**) qRT-PCR analysis of *ENO3* expression after inhibition of METTL3 in HPASMCs. (**C**) Western blot analysis and quantification of ENO3 expression after inhibition of METTL3 in HPASMCs. (**A-C**) The data were analyzed using two-tailed Student's t-test. Data are presented as mean ± SD. **P < 0.01.

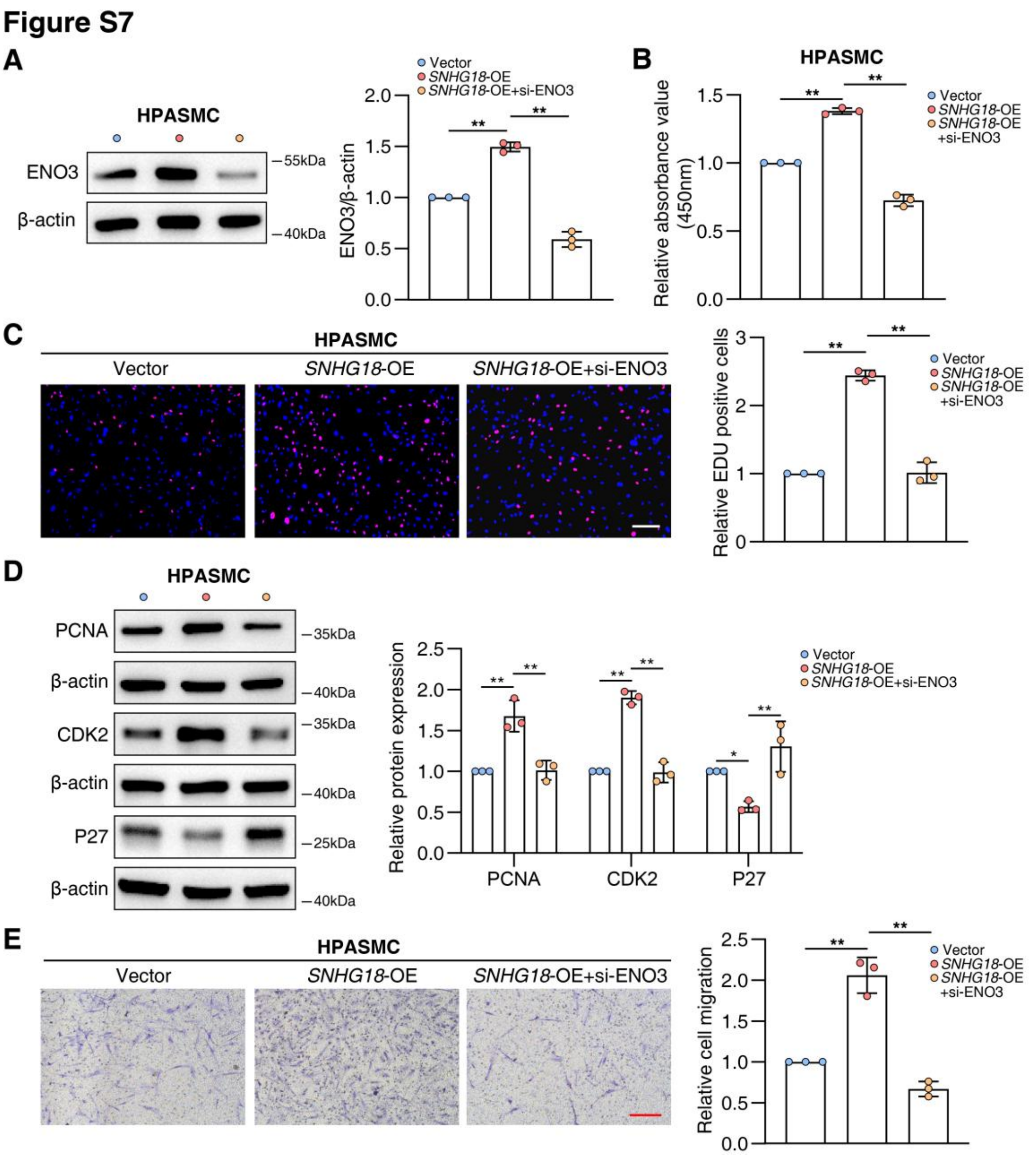

Figure S7 ENO3 attenuates *SNHG18*-mediated HPASMC proliferation. (**A**) Western blot analysis and quantification of ENO3 expression in HPASMCs. (**B**) CCK8 assays determined the proliferation of HPASMCs after indicated treatments. (**C**) EDU assays determined the proliferation of HPASMCs after indicated treatments. Scale bar 200 μm. (**D**) Western blot analysis and quantification of PCNA, CDK2, and P27 expression in HPASMCs. (**E**) Transwell assays determined the migratory ability of HPASMCs after indicated treatments. Scale bar 200 μm. (**A-E**) The data were analyzed

using one-way ANOVA with Dunnett's multiple comparisons test. Data are presented as mean ± SD. *P < 0.05, **P < 0.01. OE indicates overexpression.

Figure S8

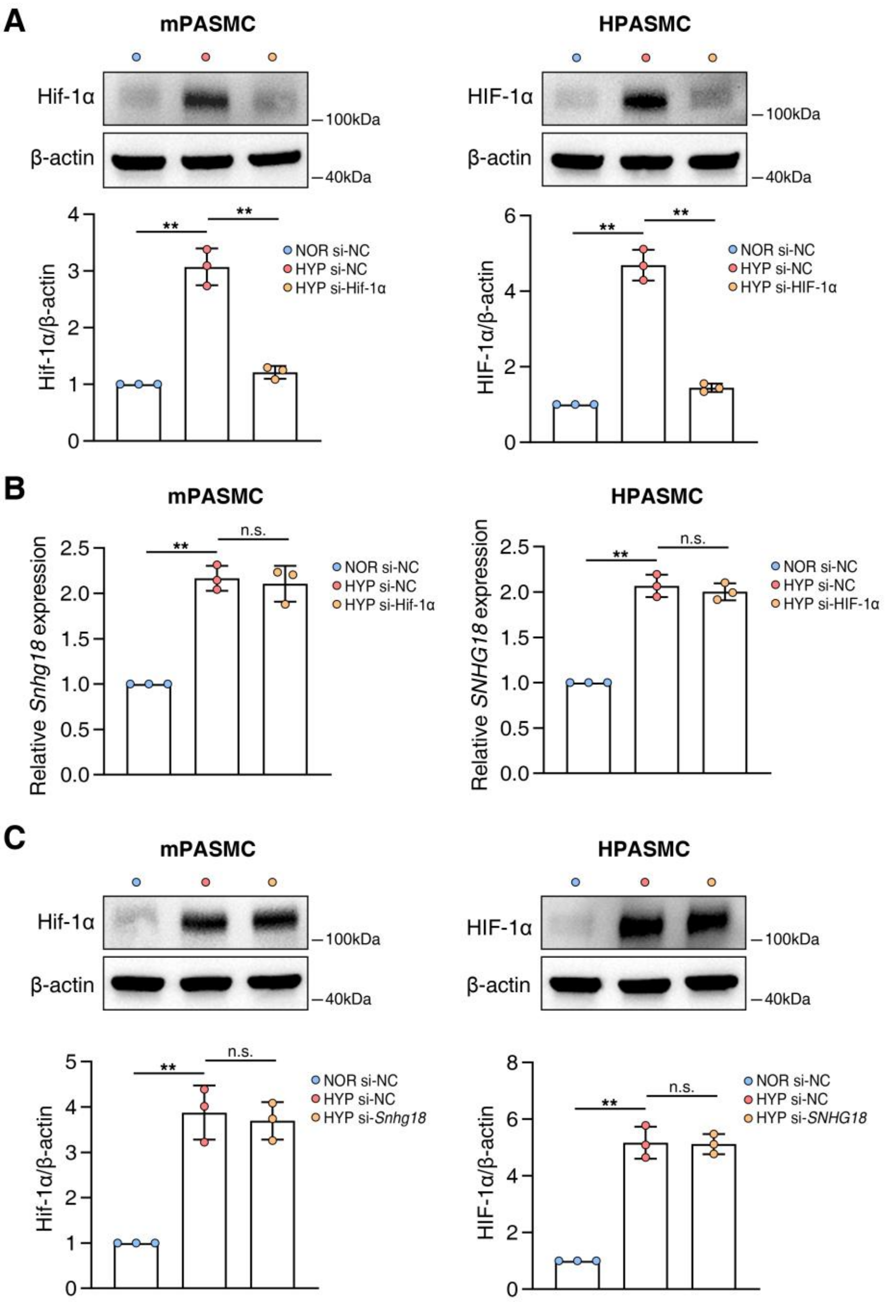

Figure S8 The relationship between HIF-1α and *Snhg18*. (**A**) The efficiency of Hif-1α knockdown was validated by Western blot analysis in mPASMCs and HPASMCs. (**B**) qRT-PCR analysis of *Snhg18* expression after Hif-1α knockdown in mPASMCs and HPASMCs. (**C**) Western blot analysis and quantification of Hif-1α expression after *Snhg18* knockdown in mPASMCs and HPASMCs. Data are presented as mean ± SD. ***p*<0.01. n.s., not significant. NOR indicates normoxia; and HYP, hypoxia.

Figure S9

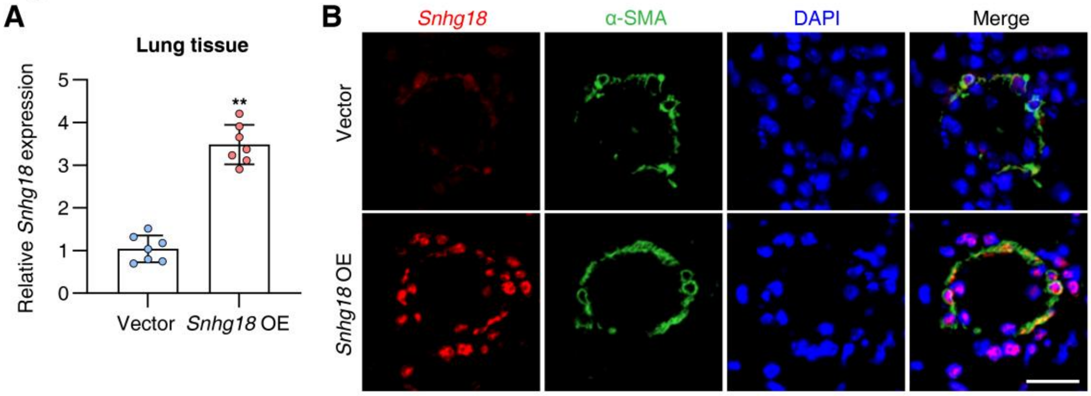

Figure S9 The efficiency of *Snhg18* overexpression in vivo. (**A**) qRT-PCR analysis of *Snhg18* expression in the mouse lung tissues (n=7). (**B**) RNA FISH-IF staining of PAs for *Snhg18* (red), α-SMA (green), and nuclei (blue). Scale bar 20 μm. (**A**) The data was analyzed using two-tailed Student's t-test. Data are presented as mean ± SD. **P < 0.01. OE indicates overexpression, and DAPI, 4′,6-diamidino-2-phenylindole.

Table S1:A list of potential Snhg18-interacting protein candidates in mPASMCs based on RNA-protein pull-down assays and mass spectrometry analysis					
**Name**	**Score**	**Peptides**	**Unique peptides**	**Coverage [%]**	**MW(kDa)**
Hnrnpa2b1	49.756	7	7	15.7	40.419
Hspa8	47.601	7	6	13.1	68.778
Tpm1	45.597	7	0	20.6	37.412
Hnrnpk	45.176	7	7	16.6	48.562
Ncl	44.594	7	7	9.9	76.722
Fn1	43.985	7	7	3	249.55
Cmtr1	43.459	7	7	8.8	96.524
Ckap4	41.644	6	6	11.5	63.691
Atxn2l	41.629	7	7	6.6	105.44
Gemin5	41.439	7	7	5.7	166.52
Dhx9	39.155	6	6	4.3	149.62
Vars1	38.664	5	5	5.1	140.21
Actg1	38.497	6	6	17.1	41.792
Msn	38.454	6	5	9.7	67.766
Hnrnph1	37.853	5	4	13.8	51.217
Hnrnpa1	37.729	5	5	14.5	38.833
Eef1a1	37.423	6	6	13.2	50.113
Iars1	37.339	6	6	4.5	144.27
Rrbp1	35.482	6	6	4	158.39
Top2a	35.056	6	6	4.5	172.79
Ddx17	34.928	7	4	12	72.584
Hnrnpd	34.426	5	5	17.2	38.354
Rps3	33.327	5	5	24.3	26.674
Map3k5	33.274	3	3	3.6	153.58
Sf1	33.14	5	5	8.9	59.698
Mars1	31.702	5	5	5.6	102.37
Yars1	30.783	5	5	8.9	63.001
Serbp1	30.742	4	4	34.7	16.078
Txlna	30.556	5	5	9.6	62.368
Septin9	30.416	5	5	9	63.772
Eif3a	29.358	5	5	3.1	161.93
Anln	28.55	4	4	4.4	122.79
Atxn2	28.511	4	4	5.3	100.09
Hnrnpab	28.346	4	4	14.4	30.831
Sub1	28.246	4	4	37	14.427
Eef1d	27.373	4	4	25.4	21.478
Septin7	26.896	4	4	9.8	50.68
Eef1g	26.239	4	4	8.2	50.06
Rpl26	26.147	4	4	17.2	17.258
Nufip2	25.966	4	4	6.8	75.656
Rbm26	25.701	4	4	4.6	111.01
Hspa5	25.603	5	4	8.7	72.421
Smc1a	25.506	4	4	3.2	143.23
Rps11	25.497	4	4	22.3	15.198
Syncrip	25.492	4	4	8.2	58.751
P4hb	25.217	4	4	8.3	57.058
Hnrnpl	25.038	4	4	7	66.821
Rps3a	24.525	4	4	14.8	29.885
Eif2s3x	24.178	4	4	9.7	51.065
Abcf2	24.084	4	4	6.1	71.781
Hsp90ab1	23.97	4	4	5.5	83.28
Dars1	23.848	4	4	9.4	57.147

Qars1	23.634	4	4	4.7	85.054
Eif2s2	23.105	4	4	9.4	38.092
Ddx1	22.937	4	4	4.5	82.499
Eif4b	22.74	3	3	8.2	68.839
Caprin1	22.069	3	3	4.7	78.168
Ktn1	21.17	3	3	2.8	137.63
Rbm3	20.786	3	3	21.6	16.604
Ddx20	20.754	3	3	4.7	91.709
Ybx1	19.821	3	3	26.6	24.74
Hnrnpf	19.687	4	3	11.3	45.729
Lars1	19.631	3	3	2.7	134.19
Cnbp	19.574	3	3	20.5	18.799
G3bp2	19.274	2	2	4.4	54.087
G3bp1	19.027	3	3	8.6	51.828
Rps18-ps6	18.999	3	3	19.1	17.671
Nono	18.935	3	3	5.1	54.54
Phldb2	18.876	3	3	2.6	127.17
Eif4g2	18.688	3	3	4.4	97.888
Mtdh	18.561	3	3	8.4	43.268
Ilf3	18.538	3	3	4.7	76.347
Lrpprc	18.452	3	3	2.3	156.61
Rps15a	18.436	3	3	29.5	8.8195
Pa2g4	18.433	3	3	6.3	43.698
Farp1	18.401	3	3	3.4	118.87
Cavin1	18.369	3	3	8.2	43.953
Ctnnd1	18.323	3	3	3.7	96.871
Matr3	18.097	3	3	7.5	42.479
Rbms2	18.034	3	3	11	37.583
Puf60	17.979	3	3	4.6	60.248
Prrc2c	17.861	3	3	1.3	302.43
Ptbp1	17.846	3	3	6.7	52.63
Clasp1	17.816	3	3	2.6	160.23
Bub3	17.804	3	3	8	36.766
Rps2	17.703	3	3	10.8	28.601
Eif3h	17.671	3	3	10.5	39.832
Edf1	17.667	3	3	16.9	16.369
Rps7	17.657	3	3	12.9	22.127
Smc3	17.572	3	3	2.3	141.55
Col5a1	17.237	3	3	1.4	183.67
Col5a2	16.441	3	2	2.3	145.02
Taf15	16.187	2	1	9	33.603
Igf2bp1	15.341	2	2	5	63.45
Dhx36	14.755	2	2	2.4	113.88
Drg1	14.73	2	2	6.8	40.512
Col3a1	14.684	2	2	2.5	138.94
Nelfb	14.427	2	2	4.4	70.32
Fmr1	14.203	2	2	3.9	66.169
Snrpd2	14.137	2	2	16.9	13.527
Strap	14.037	2	2	7.7	38.442
U2af1	13.898	2	2	8.8	27.815
Nfic	13.874	2	1	5	51.297
Rtcb	13.84	2	2	4.4	55.249
Snrpf	13.826	2	2	24.4	9.7251
Rps14	13.821	2	2	15.9	16.273
Tpm3	13.684	5	2	21.9	28.723
Eif2s1	13.673	2	2	7.9	36.108

Hnrnpa3	13.67	2	2	6	34.476
Eif3e	13.562	2	2	4.5	52.22
Sec61b	13.531	2	2	26	9.9583
Srsf3	13.526	2	2	12.2	19.329
Ywhaz	13.499	2	2	15.7	19.064
Eif3g	13.486	2	2	7.2	35.638
Rps28	13.237	2	2	35.7	6.3442
Cobll1	13.231	2	2	1.6	133.27
Prrc2a	13.18	2	2	1.4	219.46
Dnaja2	13.105	2	2	4.9	45.745
Cdc42	13.053	2	2	11	21.258
Gm8797	13.048	2	2	37.7	8.7279
Pcbp1	12.982	2	2	5.6	37.497
Ybx3	12.883	2	2	8.9	38.813
Smc2	12.814	2	2	1.8	134.24
Eif3i	12.778	2	2	6.5	36.46
Ldha	12.776	2	2	7	34.599
Rars1	12.767	2	2	3	75.673
Rpsa	12.765	2	2	22.6	10.125
Fubp3	12.761	3	2	15.7	20.651
Anxa2	12.761	2	2	11.9	19.596
Cpsf7	12.742	2	2	4.2	52.01
Pgam5	12.68	2	2	11.7	20.294
Epb41l2	12.656	2	2	2.3	101.81
Snrpb2	12.624	2	2	14.7	16.009
Sf3b2	12.609	2	2	7.8	33.162
Impdh2	12.574	2	2	3.9	55.814
Ewsr1	12.562	2	2	4.7	64.988
Aimp1	12.539	2	2	8.5	35.166
Eif3l	12.537	2	2	3.4	66.612
Elavl1	12.397	2	2	5.2	36.169
Supt16	12.323	2	2	1.9	119.84
Rps19	12.322	2	2	13.8	16.085
Gatad2b	12.263	1	1	4.2	28.659
Sf3b3	12.25	2	2	1.6	135.55
H1-2	12.247	2	2	10.8	21.266
Nudt21	12.244	2	2	7	26.24
Pspc1	12.203	2	2	3.8	58.758
Cct5	12.165	2	2	3.3	59.623
Rps13	12.138	2	2	12.1	16.142
Ckap5	12.097	2	2	1.3	218.71
Lrrc59	12.094	2	2	6.2	34.877
Tpm1	12.081	7	0	27.8	28.556
Rps4x	12.08	2	2	7.2	29.597
Rdx	12.064	4	2	6	68.542
Ap2b1	12.028	2	2	2.1	104.83
Kif2a	11.964	2	2	2.6	83.86
Nsun2	11.911	2	2	2.6	81.373
Dclk1	11.858	2	2	5	40.38
Rps5	11.854	2	2	9.3	20.413
Rps25	11.801	2	2	20.4	10.309
Parp12	11.767	2	2	3.4	79.916
Obsl1	11.763	2	2	0.9	197.96
Slc25a4	11.752	2	2	5.7	32.904
Eif3b	11.736	2	2	3.9	91.369
Fxr1	11.709	2	2	4	51.014

Usp10	11.574	2	2	2.3	87.021
Psip1	11.493	2	2	3.2	59.696
Rpl35a	11.453	2	2	14.5	12.554
Hspd1	11.426	2	2	3.1	60.955
Ubap2	11.388	2	2	2.5	117.83
Ccar1	11.366	2	2	1.4	132.06
Srsf2	11.36	2	2	6.8	25.476
Rps10	11.341	2	2	10.7	19.253
Hnrnpm	11.311	2	2	2.3	77.648
Upf1	11.307	2	2	2	123.97
Eef1b2	11.302	2	2	8.7	20.136
Acly	11.247	2	2	1.5	120.79
Eif4a2	8.8853	1	1	4.3	36.711
Otud4	8.1825	1	1	1.4	122.93
Rfc3	8.0591	1	1	3.9	40.526
FAM120A	7.8837	1	1	1.5	121.64
Hnrnpa0	7.7534	1	1	4.6	30.53
Fus	7.6654	2	1	20	13.897
Slirp	7.5491	1	1	11.8	11.444
Myl6	7.4639	1	1	9.4	15.448
Eps8	7.3638	1	1	1.7	91.736
Rps20	7.3138	1	1	9.2	13.373
Cpsf6	7.3073	1	1	2.5	59.308
Tardbp	7.2927	1	1	7.4	16.629
Rps21	7.2812	1	1	12	9.1413
Ywhae	7.2453	1	1	4.3	29.174
Cct6a	7.1423	1	1	1.9	58.076
Micu1	7.1039	1	1	5.6	26.718
Hnrnpll	7.0787	1	1	4.5	35.346
Nudt16l1	7.063	1	1	5.2	23.414
Eefsec	6.9906	1	1	10.4	13.24
Zfp207	6.9588	1	1	3.5	35.072
Sumo3	6.957	1	1	25.5	5.2631
Sf3b5	6.955	1	1	17.4	10.119
Chd4	6.9536	1	1	0.7	216.37
Ppib	6.9138	1	1	4.2	23.713
Slc16a1	6.8984	1	1	2.6	53.267
Trim25	6.8504	1	1	1.9	70.753
Zc3h4	6.8272	1	1	1.1	127.45
Fkbp3	6.7983	1	1	4.9	25.147
Rps17	6.7583	1	1	5.9	15.524
U2af2	6.7135	1	1	1.7	53.12
Pdap1	6.7016	1	1	5.5	20.605
Srsf1	6.6515	1	1	11.3	11.501
Erc1	6.6491	1	1	3.9	33.253
Snrpd3	6.6296	1	1	7.9	13.916
Sf3b1	6.6041	1	1	0.8	145.83
Ppp2r1a	6.5956	1	1	1.7	65.322
Rpl22	6.5822	1	1	8.6	14.759
Gtf2i	6.5586	1	1	6.7	16.881
Phgdh	6.5493	1	1	7.4	15.726
Xrn2	6.5297	1	1	1.3	108.69
Thbs1	6.5004	1	1	0.9	129.69
Rps6	6.4986	1	1	4.8	28.68
Septin11	6.4966	1	1	4.5	48.978
Srsf7	6.4953	1	1	8.8	15.763

Tuba1c	6.4907	1	1	2.2	49.909
Zc2hc1a	6.4797	1	1	3.7	35.152
Nfix	6.4653	2	1	4.7	53.414
Nmt1	6.4642	1	1	2.4	56.888
Znf706	6.4228	1	1	15.8	8.4977
Rfc4	6.3985	1	1	4.3	25.824
Pcbp2	6.3472	1	1	6.1	18.972
Ythdf1	6.345	1	1	1.5	63.899
1700009N14Rik	6.3343	1	1	5.1	24.357
H1-1	6.3305	1	1	5.2	21.785
Ddx6	6.3153	1	1	2.3	54.191
Rpl31	6.3079	1	1	9.2	9.9416
Rps15	6.3026	1	1	10.2	13.742
Golim4	6.2852	1	1	1.6	79.904
Sec61a1	6.2816	1	1	2.3	52.264
Cfl1	6.2666	1	1	6.2	19.714
Prpf8	6.2666	1	1	0.5	273.61
Srrt	6.2628	1	1	3.7	29.93
Pkm	6.2526	1	1	21.6	5.5684
Hspa14	6.2396	1	1	2.2	54.65
Add1	6.2361	1	1	6.2	17.827
Apex1	6.2269	1	1	3.1	32.318
Actl6a	6.2119	1	1	2.3	47.447
Ncbp1	6.1959	1	1	1.3	91.926
Aldoart1	6.1952	1	1	3	39.284
Pabpc4	6.1898	5	1	7.2	67.852
Rack1	6.1787	1	1	3.2	35.076
Asph	6.1783	1	1	1.5	75.127
Pcx	6.1306	1	1	0.8	129.7
Tpm4	6.1299	2	1	7.7	28.467
Iqgap1	6.121	1	1	0.5	188.74
Iqcf3	6.1205	1	1	4.3	19.052
Rpl35	6.0896	1	1	8.1	14.552
Mta2	6.0888	1	1	1.5	75.029
Khdrbs1	6.0848	1	1	2.3	48.37
Add3	6.0838	1	1	1.1	78.317
Eif3c	6.0729	1	1	1.1	105.53
Ddx21	6.0729	1	1	1.2	93.55
Rpl17	6.072	1	1	5.4	21.397
Rpl19	6.0636	1	1	4.6	23.247
Eef2	6.0583	1	1	1.2	95.313
Larp4	6.0557	1	1	1.6	67.487
Rpl23	6.0462	1	1	7.1	14.865
Ppfibp1	6.0374	1	1	7.3	20.499
Lsm4	6.0341	1	1	7.3	11.072
Sptan1	6.0219	1	1	0.4	282.89
Gm5478	6.0171	1	1	2.1	57.919
Smn1	5.9954	1	1	4.2	31.254
Rps27	5.994	1	1	15.4	5.6466
Flna	5.9939	1	1	13.7	7.6415
Phb2	5.958	1	1	7.6	17.416
Snrnp70	5.9559	1	1	7.8	13.842
Prr32	5.9481	1	1	3.2	30.901
Elob	5.9322	1	1	7.8	10.109
Coro1c	5.9314	1	1	13.6	7.4946
Pdia3	5.9292	1	1	2.6	56.678

Pitpnm1	5.9153	1	1	1	134.94
Eif2a	5.9062	1	1	2.4	64.403
Pabpn1	5.898	1	1	4.9	18.225
Tagln2	5.8945	1	1	5.5	22.395
Morf4l2	5.892	1	1	15.5	6.305
Lonp2	5.8892	1	1	1.2	94.525
Qki	5.8835	1	1	2.3	37.67
Igf2bp2	5.8787	1	1	1.7	58.039
Trir	5.8703	1	1	5.2	18.376
Akap8	5.867	1	1	1.3	76.293
Rpl36a	5.867	1	1	8.5	12.441
Gapdh	5.8623	1	1	2.9	29.939
Hspe1-rs1	5.8608	1	1	9.8	10.978
Thoc2l	5.8564	1	1	0.7	182.67
Sart3	5.8549	1	1	0.9	109.62
Npm1	5.8493	1	1	3.5	28.385
Rpl12	5.843	1	1	9.1	17.804
Gemin4	5.843	1	1	1.4	120.18
Wbp11	5.8358	1	1	1.7	69.874
Mccc2	5.8354	1	1	3.3	30.177
Ptbp2	5.8258	1	1	1.9	57.488
Eif6	5.8258	1	1	17.2	6.4953
Hspa9	5.8241	1	1	1.3	73.46
Spats2	5.8158	1	1	8.8	17.298
Eef1e1	5.8106	1	1	4.6	19.859
Prdx2	5.8092	1	1	5.5	15.977
Tpm1	5.8078	6	0	20.8	32.848
Snrpc	5.786	1	1	5.7	17.042
Cct7	5.7843	1	1	2.4	55.057
Rpl30	5.7828	1	1	7	12.784
Srprb	5.7784	1	1	5.2	29.579
Cct8	5.7705	1	1	2	53.082
Trp53	5.7607	1	1	2.4	42.15
Dnaja1	5.748	1	1	4.8	23.173
Snrpb	5.7401	1	1	6.5	23.656
Rpn1	5.7339	1	1	1.8	68.527
Or5m3b	5.7277	1	1	2.6	35.478
Smarca4	5.7277	1	1	0.7	143.51
Prpf19	5.7277	1	1	1.8	55.238
Crem	5.7277	1	1	9.5	10.92
Cfap57	5.7228	1	1	0.8	144.93
Eif2ak2	5.7194	1	1	2.7	58.279
Tpm2	5.7183	4	0	14.8	32.836
Pde4b	5.7162	1	1	9.9	14.035
Camk2d	5.7154	1	1	4.4	40.677
Eif3d	5.7154	1	1	1.6	63.988
Nxf1	5.7141	1	1	1.5	70.299
Rpn2	5.7112	1	1	1.3	67.501
Lrpap1	5.7084	1	1	9.8	18.248
Igf2bp3	5.7048	1	1	2.2	63.574
Txn	5.702	1	1	8.6	11.675
Ywhag	5.6938	1	1	4	28.302
Nelfe	5.6928	1	1	2.4	42.554
Myl12a	5.6892	1	1	10.3	12.28
Crocc	5.6862	1	1	0.4	226.94
Ttn	5.6859	1	1	0.3	375.53

Spata31h-ps1	5.6567	1	1	0.3	393.85
Naca	5.6492	1	1	7	23.384
Ncbp2	5.6458	1	1	4.2	19.109
Rpl24	5.6447	1	1	5.1	17.779
Dhx8	5.6423	1	1	1.3	134.36
RTRAF	5.641	1	1	3.3	28.152
Eno1	5.6379	1	1	2.2	39.782
Kif4	5.6371	1	1	0.8	139.52
Ahsg	5.637	1	1	6.1	12.61
Ip6k2	5.6354	1	1	6	46.515
Rps9	5.6352	1	1	5.9	16.206
Lrrn2	5.6245	1	1	2.1	81.122
Nhp2	5.6241	1	1	11.1	17.247
Myt1	5.6202	1	1	1.1	86.288

Table S2: The clinical characteristics of COPD-PH patients and healthy controls.

	Control (n=30)	COPD-PH (n=30)
Age (year)	69.40±12.23	75.03±9.61
Sex (male)	19 (63.33%)	20 (66.67%)
sPAP (mmHg)	N/A	54.07±16.15
WHO functional class		
I	N/A	1 (3.33%)
II	N/A	7 (23.33%)
III	N/A	19 (63.33%)
IV	N/A	3 (10%)

COPD-PH, pulmonary hypertension secondary to chronic obstructive pulmonary disease
sPAP, systolic pulmonary artery pressure

Table S3: Oligonucletides sequences summary

Gene	Forward primer	Reverse primer
Sequences of qRT-PCR primers		
Mouse		
Snhg18	GATTGTCTGACCCTGCCCAT	TAAAACCGAAGCAGCACCGA
Hnrnpa2b1	CAGGGTAGTTGAGCCAAAACG	TTCCAGACTGCCTATCGGTAA
Eno3	CACAGCCAAGGGTCGATTCC	CCCAGGTATCGTGCTTTGTCT
β-actin	GGCTGTATTCCCCTCCATCG	CCAGTTGGTAACAATGCCATGT
18S	CATTCGAACGTCTGCCCTATC	CCTGCTGCCTTCCTTGGA
Human		
SNHG18	CTGGTCAGAGGGTTGCACTT	TGAACTTGGCTGTGAGGTGG
HNRNPA2B1	ATTGATGGGAGAGTAGTTGAGCC	AATTCCGCCAACAAACAGCTT
ENO3	TATCGCAATGGGAAGTACGATCT	AAGCTCTTATACAGCTCTCCGA
β-actin	CATGTACGTTGCTATCCAGGC	CTCCTTAATGTCACGCACGAT
Sequences of siRNAs		
Mouse		
si-*Snhg18*	CCUGGCAUAUGUUACAGUAACCAUUTT	
si- Sp1	CCAAUGCCAAUAGUUAUUCAATT	
si-Hnrnpa2b1	AAGAAAGUUUGAGAAACUA	
si-Eno3	ACCGAGAAUAAGUCCAAGUUUTT	
si-Mettl3	CAAGGAAGAGTGCATGAAA	
si-Hif-1α	CCAUGUGACCAUGAGGAAATT	
Human		
si-*SNHG18*	CCACTTGGATTTCACCAAA	
si-SP1	CAGCUUGGUAUCAUCACAATT	
si-HNRNPA2B1	GAGGTGGTTATGACAACTA	
si-ENO3	CCAACAUCCUGGAGAACAATT	
si-METTL3	GCUACCUGGACGUCAGUAUTT	
si-HIF-1α	GAAGGAACCTGATGCTTTA	
Sequences of shRNAs		
sh*Snhg18*	CCTCAGCAGTAAATATCTGCA	
Sequences of ChIP primers		
Snhg18 -promoter	GTGAGCGGGCCCAGAG	GAGTGTTTGGGGGTCCCATC
